# Climate change threatens crop diversity at low latitudes

**DOI:** 10.1038/s43016-025-01135-w

**Published:** 2025-03-04

**Authors:** Sara Heikonen, Matias Heino, Mika Jalava, Stefan Siebert, Daniel Viviroli, Matti Kummu

**Affiliations:** 1 Aalto University, Department of Built Environment, Espoo, Finland; 2 University of Göttingen, Department of Crop Sciences, Göttingen, Germany; 3 University of Zürich, Department of Geography, Zürich, Switzerland

**Keywords:** Climate-change impacts, Agriculture, Climate-change adaptation

## Abstract

Climate change alters the climatic suitability of croplands, likely shifting the spatial distribution and diversity of global food crop production. Analyses of future potential food crop diversity have been limited to a small number of crops. Here we project geographical shifts in the climatic niches of 30 major food crops under 1.5–4 °C global warming and assess their impact on current crop production and potential food crop diversity across global croplands. We found that in low-latitude regions, 10–31% of current production would shift outside the climatic niche even under 2 °C global warming, increasing to 20–48% under 3 °C warming. Concurrently, potential food crop diversity would decline on 52% (+2 °C) and 56% (+3 °C) of global cropland. However, potential diversity would increase in mid to high latitudes, offering opportunities for climate change adaptation. These results highlight substantial latitudinal differences in the adaptation potential and vulnerability of the global food system under global warming.

## Abstract (web summary)

The impacts of shifting climate suitability on current crop production, particularly how this might change food crop diversity, remain understudied. This study assesses the future climatic suitability of global croplands for 30 major food crop types, quantifying the changes in potential food crop diversity given climate conditions across four global warming levels.

## Main

Climate change threatens global food security and has already impacted the productivity of major food crops^1^ and geographically shifted cropping areas^2^. Future projections indicate that increasing temperatures and changing precipitation patterns will decrease the yields of staple crops, especially at low latitudes, whereas agriculture in temperate regions could benefit from warmer average conditions^3–6^. It has been estimated that by 2100, up to 30% of global food crop production could experience climate conditions that currently do not host major crop production anywhere across the globe^7^. Although the existing research on climate change impacts has focused mainly on four global staple crops (rice, maize, wheat and soybean)^3–6^ or several crops aggregated^7^, the projected rapid changes in climate conditions could challenge the adaptive capacity of current crop production across crop types, especially in the equatorial region^7,8^.

Changing climate conditions, together with various socio-economic factors such as market incentives, will probably influence the diversity of crop types that can feasibly be cultivated on current croplands^9–13^. Higher diversity in local crop production supports the stability and the diversity of food supply at the national scale^14–16^ and the resilience of production to stressors such as pests and the increasingly frequent adverse weather conditions due to climate change^17–20^. Furthermore, crop diversity allows climate change adaptation by selecting crops that are resilient to local climate conditions^2,13,21^ or by diversifying production (for example, through crop rotation)^13,19,22^. Several studies on the climatic suitability of croplands show that the optimal climate conditions for many food crops are shifting away from low-latitude regions^12,23–26^ towards mid to high latitudes^12,27–29^. However, these studies mostly focus on district- to regional-scale analyses^23,26–28,30^ or cover a limited number of food crops^24,25,31–33^, hence hindering quantitative comparisons of impacts between individual crops or analyses of potential food crop diversity across regions. Furthermore, existing studies analysing future changes in potential crop diversity at the global scale have focused on the environmental impacts of such changes^12,29^, rather than the consequences for climate change adaptation potential in food crop production. Thus, a comprehensive and quantitative global view of the impacts of shifting climate suitability on current crop production, and changes in potential food crop diversity, is lacking.

**Extended Data Fig. 1:**
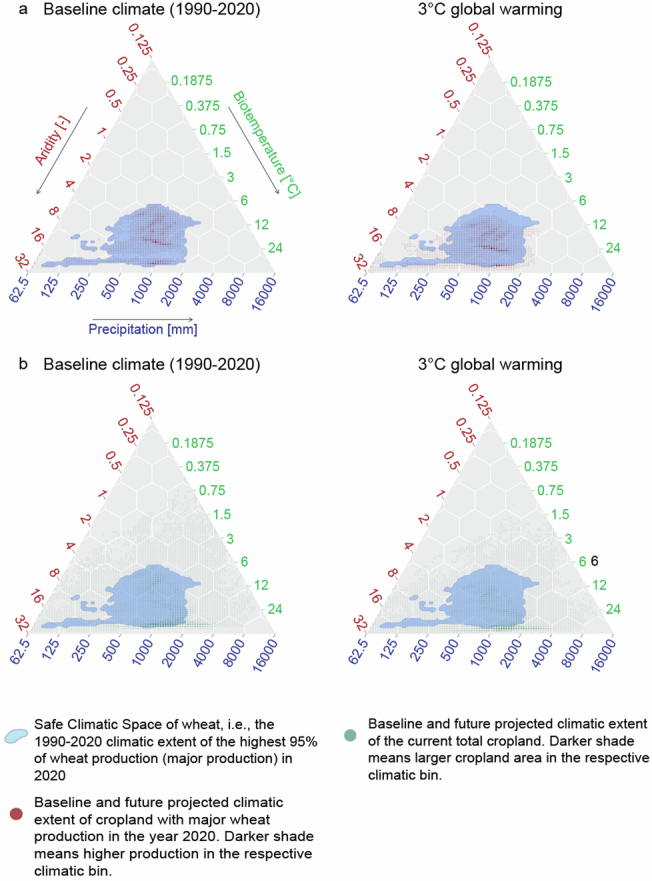
Current wheat production and total cropland area in relation to the Safe Climatic Space (SCS) of wheat. The crop-specific SCS, delineated by the light blue area, was defined based on the baseline (1990-2020) climatic extent of major wheat production in 2020 (production contributing to the highest 95% in metric tons). Panel **a**) The red circles show the climatic extent of the 2020 major wheat production in the baseline climate and under 3 °C global warming (median from eight General Circulation Models). A darker shade of red denotes higher production in the respective climatic bin (altogether, there were 1000 climatic bins^7^). The red circles, that is, production locations that overlap with the blue area, are within the SCS of wheat. Panel **b**) The green circles represent the climatic extent of the total cropland area of the 46 crop types in SPAM 2020^35^ under the baseline climate and 3 °C global warming (median from eight General Circulation Models). The green circles, that is, cropland area, that overlap with the blue area are within the SCS of wheat. A darker shade of green denotes more cropland area in the respective climatic bin. Source data

**Extended Data Fig. 2:**
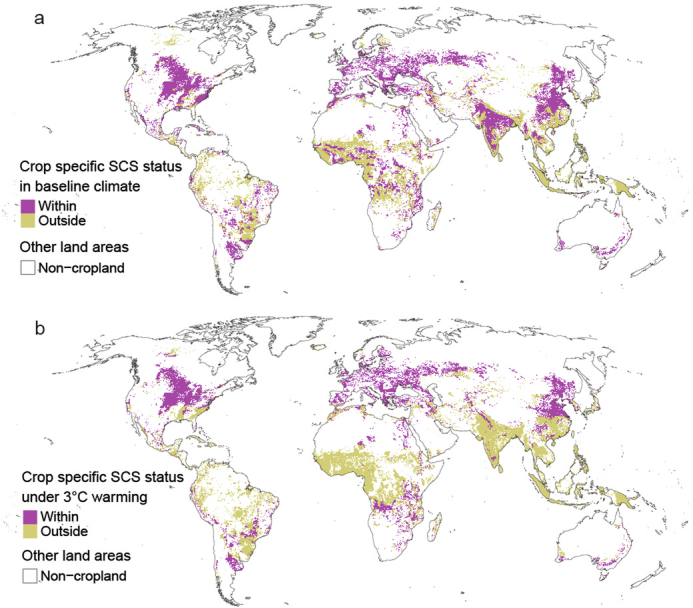
The geographical extent of the Safe Climatic Space (SCS) of wheat on the total cropland area. The total cropland area covers the cropland of all the 46 crop types in the SPAM 2020 data. Purple cropland areas are within the SCS of wheat, and yellow cropland areas are outside the SCS of wheat. Panel **a**) baseline climate, panel **b**) 3 °C global warming. In the future climate, that is, panel **b**), a grid cell is set ‘within the SCS’ if at least half of the eight General Circulation Models indicate this. Basemaps from Natural Earth (naturalearthdata.com).

To bridge this knowledge gap, we assess the future climatic suitability of global croplands for 30 major food crop types and quantify the changes in potential food crop diversity given climate conditions across four global warming levels ranging from 1.5 °C to 4 °C (Table 1)^34^. We delineate the climatic niche for each crop by applying the Safe Climatic Space (SCS) concept^7^, which maps the current climatic space of the major production areas of each crop^35^ (contributing to the largest 95% of production) using three climate parameters: annual precipitation, biotemperature and aridity^36^ (Extended Data Fig. 1). Then, under future climate conditions^36^, we examine which locations would fall outside these climatically fixed, crop-specific SCSs, both on the current production areas of each crop and on the total cropland of all crops (example for wheat in Extended Data Figs. 1 and 2). Applying the SCS concept for individual crops, instead of combined production^7^, and extending projections on the total global cropland area allows us to holistically project global changes in the climatic potential of croplands and to globally identify hotspots of increasing or decreasing climatic suitability, for example, for all cereal crops. Additionally, it allows us to analyse changes in the potential diversity of food crops, which is important for the resilience of crop production^14,15,19^, given future temperature and moisture conditions.

**Table 1:** IPCC estimates of warming level crossing year ranges following the different SSPs

Warming level	SSP	IPCC central estimate of warming level crossing (centre year)
1.5 °C	SSP1–2.6	2023–2042 (2033)
2 °C	SSP2–4.5	2043–2062 (2053)
3 °C	SSP3–7.0	2066–2085 (2076)
4 °C	SSP5–8.5	2075–2094 (2085)

SSPs: Shared Socio-economic Pathways

IPCC: Intergovernmental Panel on Climate Change

## Results

### Risk to current production under warming levels

To determine changes in the share of current crop production (in metric tons) that would fall outside crop-specific Safe Climatic Space^7^ (SCS), we projected climatic shifts on the current cultivation areas of each crop across warming levels (Methods). Then we found the current production locations of each crop where climate conditions would shift outside the crop-specific SCSs (hereafter, ‘locations or production would shift outside the SCS’) under a warming level (Extended Data Fig. 1). To locally examine the risk to the current production of all 30 analysed food crops, we identified the lowest global warming level that would push at least 25% of the current production in metric tons in a grid cell outside the crop-specific SCSs. We used this 25% threshold for indicating ‘considerable risk’ to current production. In addition, we examined globally and regionally the aggregated shares of current production that would fall outside the SCS for individual crops, all food crops in total and five crop groups (Supplementary Table 1). We tested the sensitivity of these results to three factors: the threshold used for describing considerable risk to current production (25%, 50% and 75%; Methods), the selected crop production data (here we used two previous versions of the crop production data used in the main analysis; Supplementary Note 1) and climate seasonality (here we used climate data only over the growing season instead of the full year; Supplementary Note 1).

**Fig. 1:**
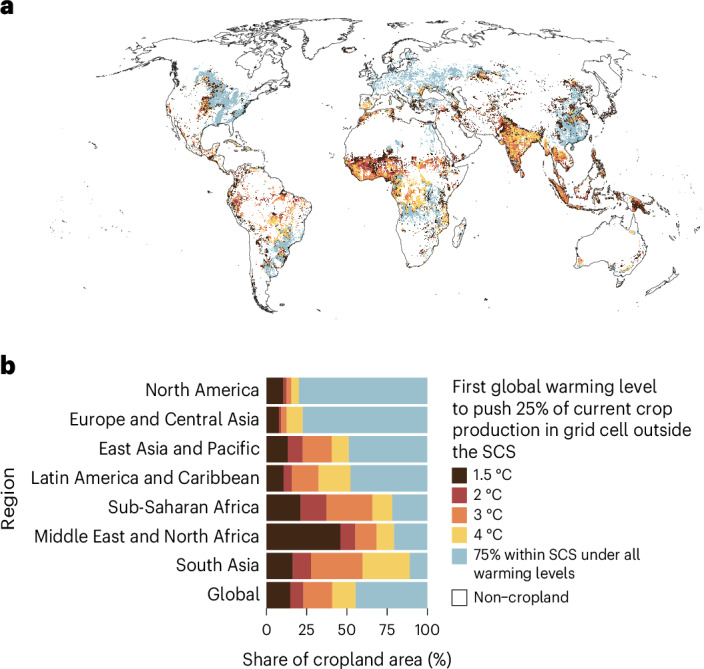
Lowest global warming level that would push current crop production into considerable risk. ‘Considerable risk’ is defined as at least 25% of the local current production of the 30 assessed food crop types (in a grid cell, in metric tons) outside crop-specific SCSs. ‘75% within SCS under all warming levels’ category indicates that in the grid cell, current production would not be pushed into considerable risk under any global warming level, that is, at least 75% of production in that location is within the crop-specific SCSs under all four warming levels. A cell is assigned within the SCS for an individual crop if at least half of the eight General Circulation Models used in future projections indicate this. **a**, Lowest global warming level to push current production in grid cell into considerable risk. **b**, Regional share of cropland area pushed to considerable risk under each warming level. The boundaries of the seven regions are shown in Supplementary Fig. 4, ‘Global’ includes all regions. Extended Data Fig. 3 and Supplementary Table 2 show similar analyses with 50% and 75% thresholds for indicating risk to current production. Basemap in **a** from Natural Earth (naturalearthdata.com). Source data

Our results show that the adverse changes in climate conditions for current food crop production are concentrated in tropics and subtropics. There the current production would be at considerable risk on large areas especially if global warming exceeds 2 °C (Fig. 1). Notably, in the Middle East and North Africa, the current crop production would be at considerable risk on nearly 50% of cropland area already under 1.5 °C global warming. Furthermore, in the Middle East and North Africa, these areas with considerable risk to current production would cover 69% of the cropland area under 3 °C global warming and in South Asia and in sub-Saharan Africa, 60%. In contrast, especially in the Northern Hemisphere, there are large areas where the current production would not face considerable risk under any studied warming level. These lower risk areas cover 80% of the croplands in North America and 77% of those in Europe and Central Asia.

**Extended Data Fig. 3:**
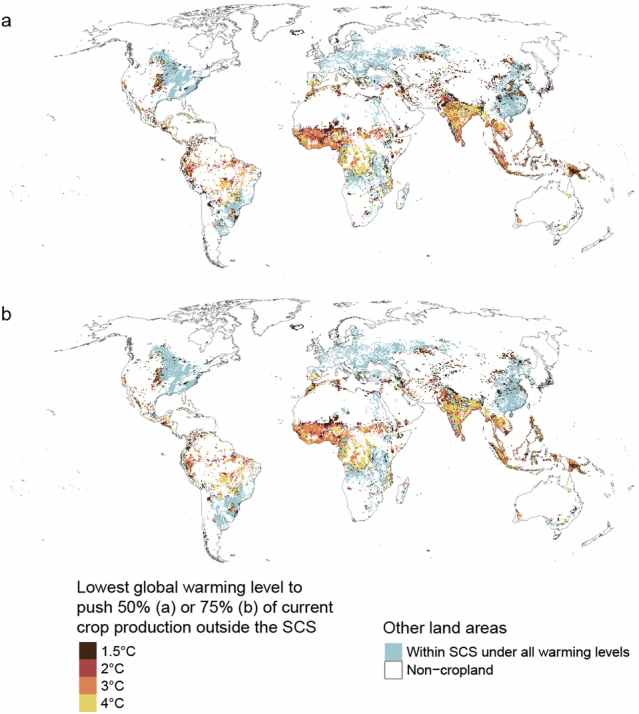
The lowest global warming level that would push current local crop production into considerable risk at risk thresholds 50% and 75%. ‘Considerable risk’ is defined as at least 50% (panel **a**) or 75% (panel **b**) of the local current production of the 30 food crops (in a grid cell, in metric tons) outside crop-specific Safe Climatic Spaces (SCS). “Within SCS under all warming levels” category indicates that in the grid cell, current production would not be pushed into considerable risk under any warming level, that is, at least **a**) 50% and **b**) 25% of production in that location is within the crop-specific SCS under all four global warming levels. A cell is assigned within the SCS for an individual crop if at least half of the eight General Circulation Models used in a future projection indicate this. Basemaps from Natural Earth (naturalearthdata.com).

In our sensitivity analysis, using three thresholds instead of only 25% for describing considerable risk to current production, the results remained consistent across the percentage thresholds in regions facing relatively small adverse effects, whereas in regions that would experience the most severe adverse effects, the analyses using the thresholds of 50% and 75% revealed relatively milder risks to current production (Extended Data Fig. 3 and Supplementary Table 2). This could indicate that in the less affected regions, locally, the currently cultivated crops share similar climatic niches, whereas in the more severely affected regions, the currently cultivated crops have more diverse climatic niches. Furthermore, we tested the sensitivity of these results to the crop production data used for delineating crop-specific SCSs (SPAM 2020^35^ compared with its previous versions: SPAM 2005^37^ and SPAM 2010^38^; Supplementary Note 1 and Supplementary Tables 7 and 8), and the results were mostly similar to the main results in Fig. 1.

### Current production shifting outside the SCSs

**Fig. 2:**
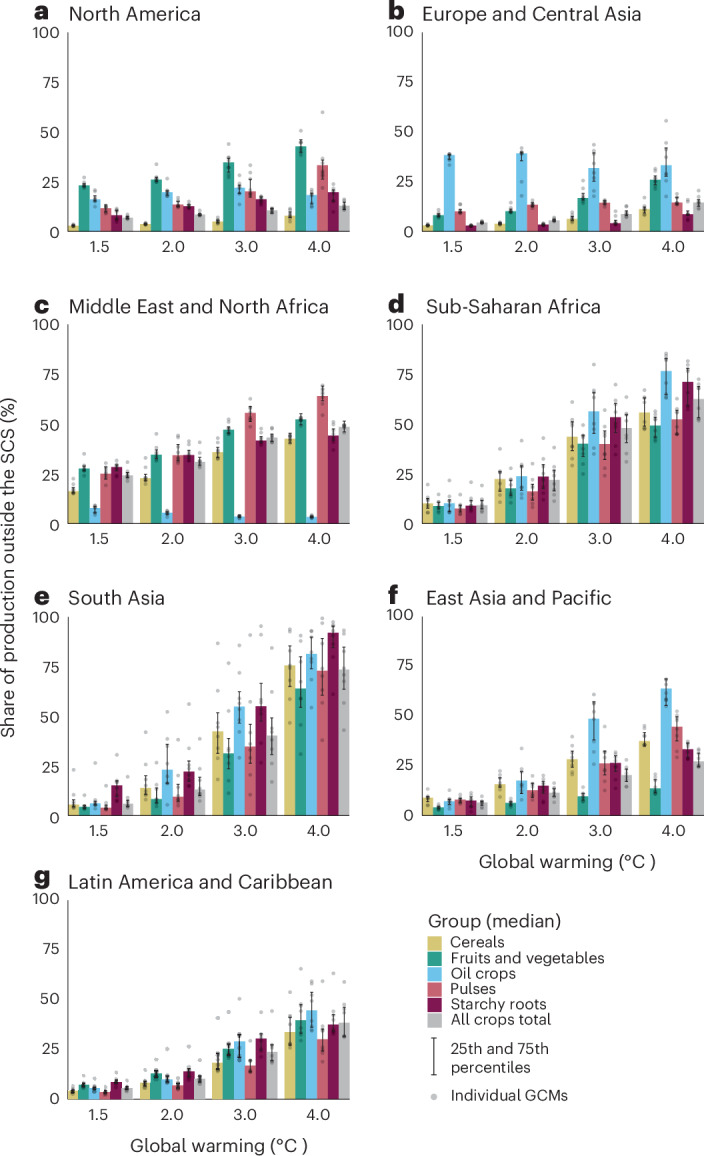
Regional share of current crop production falling outside the SCSs in crop groups. The production volume (in metric tons) outside the crop-specific SCS was calculated individually for the 30 analysed food crop types in seven regions (Supplementary Fig. 4), with estimates from eight General Circulation Models (GCMs) and then aggregated into the share of production outside the SCS within crop groups. The classification of crop types into crop groups is shown in Supplementary Table 1. In the bar charts, the bar height shows the crop group median estimate from the eight GCMs and the whiskers show the 25th and 75th percentiles of the estimates. The grey dots show estimates from individual GCMs. **a**–**g**, Regions. Source data

At the level of crop groups (Supplementary Table 1) and for food crops in total, in several regions, the aggregated share of current crop production that would fall outside the crop-specific SCSs would be moderate (less than 25%) under 1.5–2 °C global warming but would increase steeply if global warming exceeded 2 °C (Fig. 2). However, in the Middle East and North Africa, 31% of the total crop production would shift outside the SCS already under 2 °C global warming. In addition, regionally, there are crop groups in which more than 25% of the current production would be pushed outside the SCS already under 2 °C warming: fruits and vegetables in North America (26%) and in the Middle East and North Africa (35%), oil crops in Europe and Central Asia (39%) and pulses and starchy roots in the Middle East and North Africa (34% in both crop groups).

**Extended Data Fig. 4:**
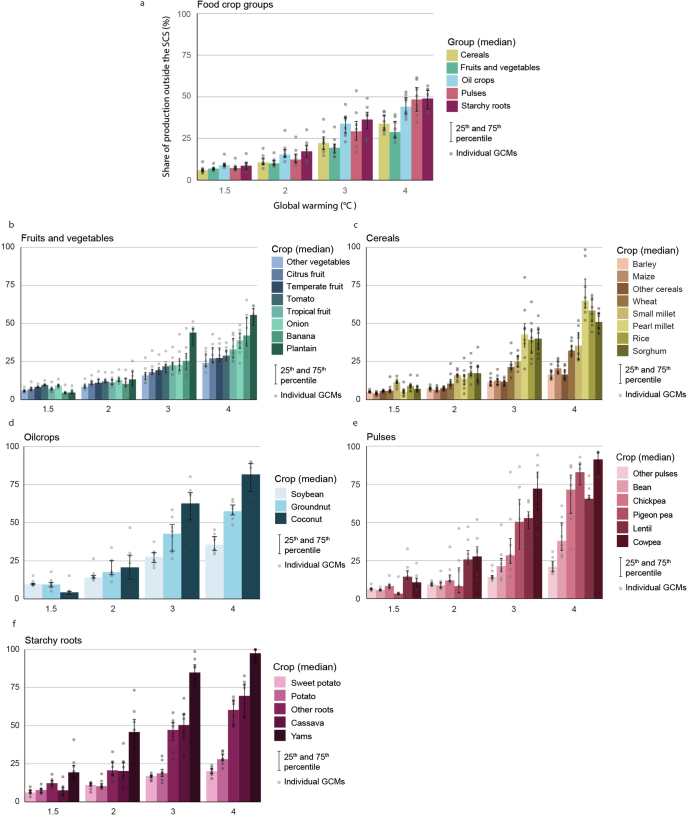
The share of current global food crop production outside the crop-specific Safe Climatic Space (SCS) under global warming. The share of production outside the SCS was calculated individually for the 30 crops from production data in metric tons, with estimates from eight General Circulation Models (GCM). The crop group-level results for five crop groups are aggregated from the results for individual crops. Bar height shows the median estimate from the GCMs, whiskers show the 25^th^ and 75^th^ percentiles of the estimates, and grey dots show estimates from individual GCMs. Panel **a**): crop groups (see Supplementary Table 1), panels **b**) – **f**): individual crops. Source data

For most individual crops, as for crop groups, less than 25% of the aggregated current global production would be pushed outside the crop-specific SCSs with 1.5–2 °C global warming (Extended Data Fig. 4), but the share of production at risk would steeply increase if warming exceeded 2 °C. Globally, the largest adverse effects are seen for those crops whose current production^35^ is centred in a relatively small area, covering a relatively small variety of climate conditions near the Equator. These crops have relatively narrow SCSs, and their current production areas are projected to face globally compared hot and arid climate conditions. The four crops facing the largest adverse effects on current production are coconut, yams, cowpea and pigeon pea. For these four crops, 50% or more of the current global production would fall outside of the SCS under 3 °C global warming and more than 75% under 4 °C warming. Rice, which is also cultivated mainly in the equatorial region^35^, would be among the most adversely impacted cereal crops under all warming levels, with 17% of the current production outside the SCS already under 2 °C warming. On the other hand, for crops whose production is centred in mid to high latitudes (for example, barley, sweet potato) and crops whose production is distributed widely across current croplands and a variety of climates (for example, maize), the adverse effects for current production would remain moderate (<25% current production outside the SCS), even under 4 °C global warming (Extended Data Fig. 4). The staple crops wheat and soybean have important production regions both in the equatorial region and at mid to high latitudes^35^, which is reflected in more than 25% of current production being pushed outside the SCS under higher than 2 °C warming (soybean) and higher than 3 °C warming (wheat). We tested the sensitivity of the results for soybean and maize to climate seasonality and found similar changes in production shifting outside the crop-specific SCSs (Supplementary Table 14 and Supplementary Table 15), as in the main results in Extended Data Fig. 4.

### Changes in total cropland within the SCSs

After examining the current production areas of each of the 30 food crops, we expanded the analysis beyond the cultivation areas of individual crops to cover the current total, combined cropland of all crops (Methods). This allowed analysis of the climatic potential of all current croplands for each of the 30 food crops, regardless of their current cultivation areas. Here we determined changes in the total geographical cropland area where climate conditions would be within the crop-specific SCS (that is, cropland is ‘within the SCS’) from the baseline climate to the projected future climate (example for wheat in Extended Data Figs. 1 and 2). We tested the sensitivity of these results to the selected crop production data and to the seasonality of climate conditions (Supplementary Note 1).

**Fig. 3:**
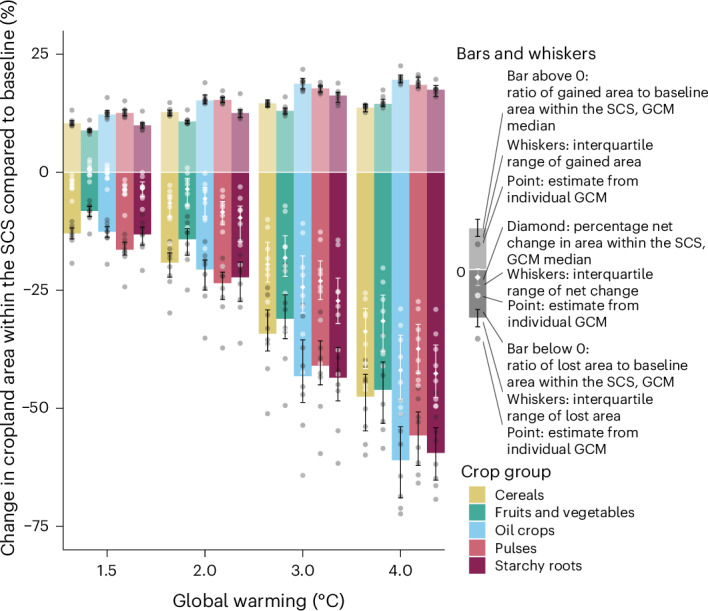
Global changes in the current total cropland area within the SCSs in crop groups. The bars show the crop group median estimates of the ratio of gained cropland area (positive values on the vertical axis) and the ratio of lost cropland area (negative values on the vertical axis) within the SCS compared with the baseline from eight GCMs, the black whiskers show the 25th and 75th percentiles of these estimates, and the grey dots show estimates from individual GCMs. The white diamond shape shows the median percentage net change in cropland within the SCS, the white whiskers show the 25th and 75th percentiles of the GCM estimates, and the white dots show estimates from individual GCMs. The area changes were first calculated for the 30 individual crop types by comparing the cropland area within the crop-specific SCSs under global warming levels from 1.5 °C to 4 °C to the total cropland area within the crop-specific SCSs under baseline climate conditions (1990–2020). Then the change in area was aggregated within five crop groups and converted to a percentage change or area ratio (Methods). The classification of crop types into crop groups is shown in Supplementary Table 1. Source data

At the level of crop groups, the global net change in cropland area within the SCS would be negative in all groups if global warming exceeded 1.5 °C (Fig. 3). The net change and the ratios of gained and lost cropland area within the SCS compared with the baseline are relatively similar in all crop groups under the same warming level. The global net change in cropland area within the SCS would be the largest for starchy roots under most warming levels (−3% to −43% under 1.5 °C to 4 °C global warming) and the smallest for fruits and vegetables (0% to −32% under 1.5 °C to 4 °C global warming). Under the higher warming levels 3 °C and 4 °C, oil crops would face the largest ratio of both gained and lost cropland area: +19% to +20% and −43% to −61%, respectively.

**Fig. 4:**
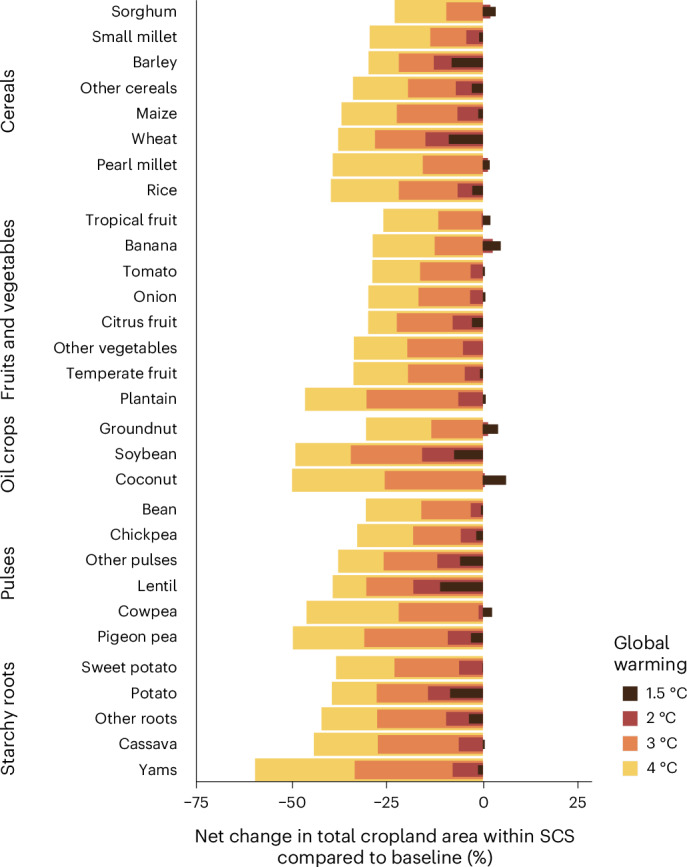
Global percentage net change in total current cropland area within the crop-specific SCSs. The percentage change is in comparison to the total cropland area within the crop-specific SCS under the baseline (1990–2020) climate conditions and values were calculated individually for 30 food crop types. The change in the total cropland area within the SCS was calculated by comparing the cropland area where climate conditions are within the crop-specific SCS under global warming levels from 1.5 °C to 4 °C to the total cropland area where climate conditions are within the crop-specific SCS under baseline climate conditions (1990–2020), regardless of the current cultivation area of each crop. The length of the nested bar shows the median percentage net change in area within the SCS under a warming level using estimates from eight GCMs. The 25th and 75th percentiles of the estimated percentage net change are shown in Supplementary Table 3. Source data

The net decrease in global cropland within the SCS is further highlighted at the level of individual food crops (Fig. 4). For more than half of the 30 analysed crop types, there would be a net decrease in cropland within the SCS already under 1.5 °C warming. If global warming exceeds 2 °C, the net change would be negative for all 30 crops. Notably, under the lower warming levels, potato and soybean would experience the largest net decreases in cropland within the SCS (Fig. 4) in their respective crop groups even though they would experience relatively small adverse impacts on current production (Extended Data Fig. 4). Moreover, the staple crops soybean, wheat, maize and rice would face some of the largest net decreases in cropland within the SCS under all warming levels compared with other cereal and oil crops (Fig. 4).

For all individual crops, we performed a sensitivity analysis using previous versions of the crop production data. For most crops, changes in cropland area within the SCS (Supplementary Table 10) remained similar to those in the main analysis (Fig. 4). Furthermore, for soybean and maize, we tested the sensitivity of the results to climate seasonality (Supplementary Table 16) and found that applying the seasonal approach affected the spatial distribution of croplands within the SCS but not the global percentage net change in cropland area within the SCS.

### Changes in potential crop diversity on the current croplands

Finally, utilizing the analysis of changes in cropland area within the crop-specific SCSs, we determined the changes in potential crop diversity under the four global warming levels. The potential diversity of food crops describes the theoretical number of food crops that could be cultivated in a location, given that the climate conditions are within the crop-specific SCSs. The change in potential diversity was measured as the percentage change in the number of crops that could be cultivated in each location on the current total cropland from baseline climate conditions (1990–2020) to a future global warming level, regardless of where each crop is currently cultivated. In addition, we identified those current cropland areas where there is currently only marginal production of any of the 30 food crops (contributing to the lowest 5% of the global total of an individual crop, in metric tons) but that would climatically shift into the SCS of at least one crop under a global warming level. These areas are referred to as ‘cropland with emerging climatic potential’. Finally, we produced maps of baseline and future total potential crop diversity, showing the theoretical number of crops that could be cultivated in each location based on the crop-specific SCS. Additionally, we tested the sensitivity of these results to the selected crop production data (Supplementary Note 1).

**Fig. 5:**
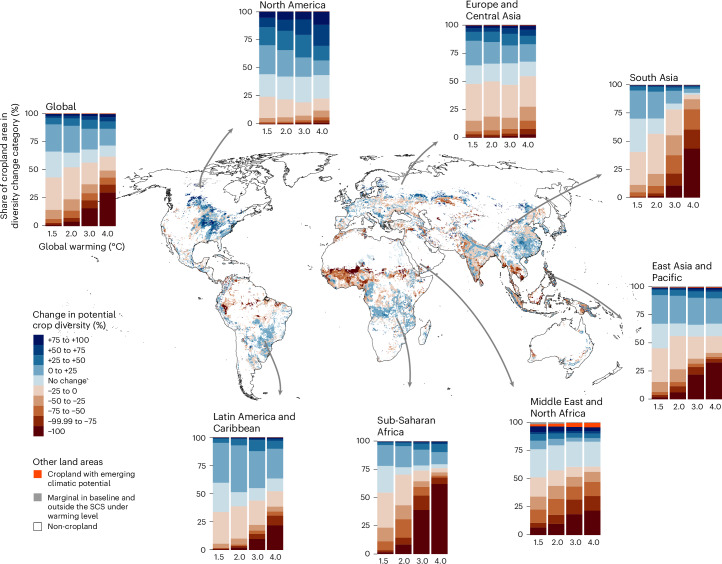
Global and regional percentage changes in potential food crop diversity. The map shows the global change in the potential diversity of 30 food crop types under 2 °C global warming, and stacked bar plots show in seven regions (Supplementary Fig. 1) and globally the share of current cropland area in categories of potential diversity change under four global warming levels from 1.5 °C to 4 °C. The change in potential diversity is measured as the percentage change in the number of crops that could be cultivated at each location given the temperature and moisture conditions, from baseline climate conditions (1990–2020) to a future global warming level. The number of crops that could be cultivated in a location is determined by the extent of cropland within the crop-specific SCSs in the baseline climate and under each warming level, regardless of the current cultivation area of each crop. For an individual crop, a single location is defined within the SCS under a warming level if at least half of the eight GCMs indicate so. ‘Marginal in baseline and outside the SCS under warming level’ indicates that the location currently hosts marginal crop production and does not shift into the SCS of any crop under the warming level. ‘Cropland with emerging climatic potential’ indicates that the location currently hosts marginal crop production but shifts into the SCS of at least one crop under the warming level. Basemap from Natural Earth (naturalearthdata.com). Source data

Globally, already under 2 °C warming, the potential crop diversity would decrease on more than half of the global cropland area (Fig. 5 and Supplementary Table 4). The most spatially extensive decline in potential crop diversity is observed near the Equator, for example, in sub-Saharan Africa and South Asia. In these regions, the potential diversity of food crops would decrease on more than 70% of the current cropland area if global warming exceeds 2 °C. In contrast, in North America, Latin America, and Europe and Central Asia, there would be an increase or no change in potential crop diversity on more than half of the cropland area under up to 3 °C global warming. The sensitivity analysis using two previous versions of the crop production dataset showed similar results (Supplementary Table 12 and Supplementary Table 13) as the main analysis (Supplementary Table 4).

**Extended Data Fig. 5:**
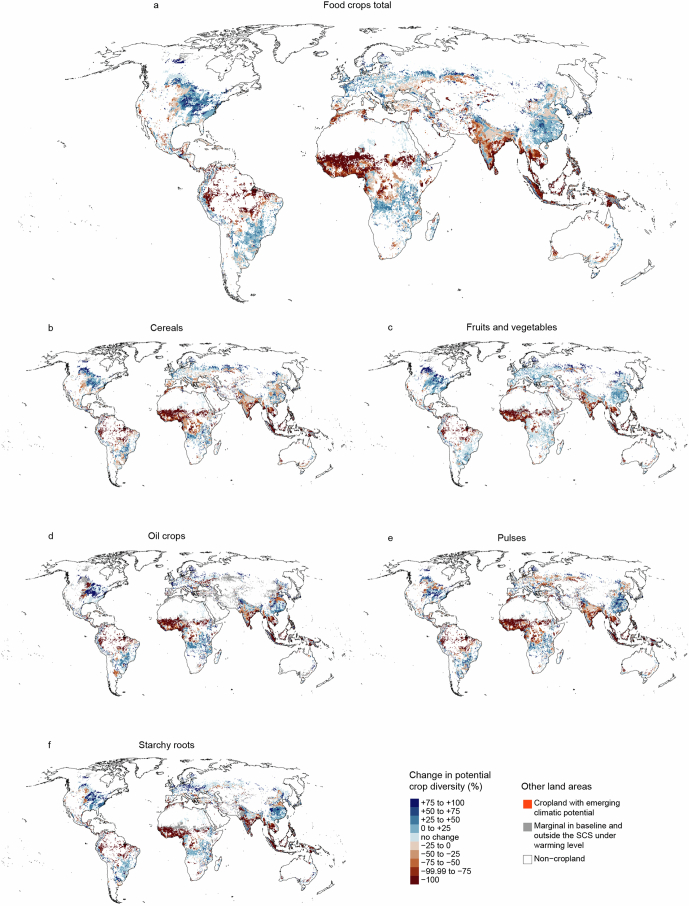
Global percentage change in potential crop diversity under 3 °C global warming. The change in the potential diversity is measured as the percentage change in the number of crops that could be cultivated at each location given the temperature and moisture conditions, from the baseline climate conditions (1990–2020) to the global warming level. The number of crops that could be cultivated in a location is determined by the geographical extent of the crop-specific Safe Climatic Spaces (SCS), regardless of the current cultivation area of each crop. For an individual crop, a location is defined within the SCS if at least half of the eight General Circulation Models indicate this. “Marginal in baseline and outside SCS under warming level” indicates that the location currently hosts marginal or no production of crops within a crop group and does not shift into the SCS of any crop in the crop group under the warming levels. “Cropland with emerging climatic potential” indicates that the location currently hosts marginal or no production of crops within a group but shifts into the SCS of at least one crop under the warming level. Panel **a**): 30 food crops total; panels **b**)–**f**): five crop groups (Supplementary Table 1). Basemaps from Natural Earth (naturalearthdata.com).

In most regions, the loss of potential diversity would become more severe with increasing global warming, while the increase would stagnate (Fig. 5 and Extended Data Fig. 5). There is a notable increase in the share of cropland in the most severe categories of decreasing potential diversity (−75% to −100%) if global warming exceeds 2 °C (Fig. 5). Notably, in the Middle East and North Africa, the most severe categories of decreasing potential diversity cover 11% of the cropland area already under 1.5 °C warming. The steepest adverse development is observed in sub-Saharan Africa, where the most severe categories of decreasing potential diversity would cover 1% of the current cropland area under 1.5 °C warming but 68% under 4 °C warming. The opposite development is seen especially in North America. There, the category with the greatest increase in potential diversity (up to +75% to +100%) covers more cropland with increasing warming. In North America, areas with a +75% to +100% increase in potential diversity would cover 6% to 12% of cropland area under 1.5 °C to 4 °C warming, respectively.

A small share (less than 1%) of the current marginal cropland shifts within the SCS of at least one crop under all warming levels (Fig. 5 and Supplementary Table 4). Although these areas are fragmented, summarization within elevation zones (Supplementary Fig. 6) reveals that relatively the largest shares of these cropland areas with emerging climatic potential are located in high elevation regions (>2,500 m), which generally become warmer and drier due to climate change^39^ and therefore climatically more favourable for cultivating food crops.

**Fig. 6:**
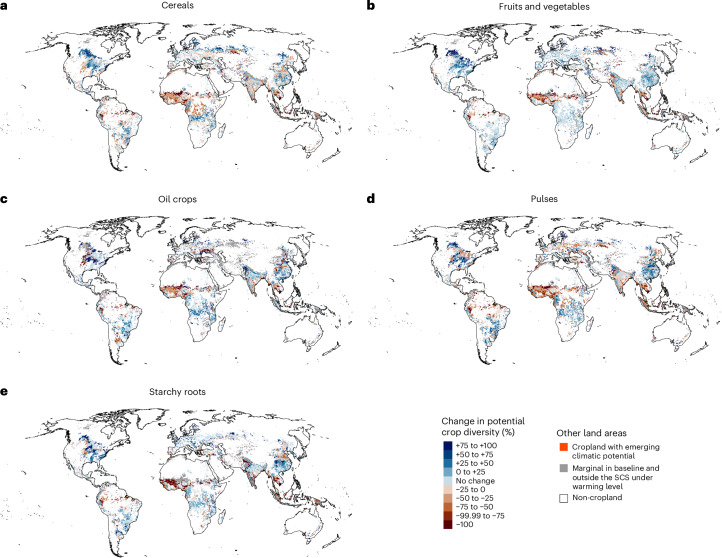
Global percentage change in potential crop diversity within crop groups under 2 °C global warming. The change in the potential diversity is measured as the percentage change in the number of crops that could be cultivated in each location given temperature and moisture conditions, from baseline climate conditions (1990–2020) to a future global warming level. The number of crops that could be cultivated in a location is determined by the geographical extent of the crop-specific SCS in the baseline climate and under warming levels, regardless of the current cultivation area of each crop. The analysis covered 30 crop types, classified into five crop groups (Supplementary Table 1). For an individual crop, a location is defined within the SCS under a warming level if at least half of the eight GCMs indicate this. ‘Marginal in baseline and outside SCS under warming level’ indicates that the location currently hosts marginal or no production of crops within a crop group and does not shift into the SCS of any crop in the crop group under the warming level. ‘Cropland with emerging climatic potential’ indicates that the location currently hosts marginal or no production of crops within a group but shifts into the SCS of at least one crop under the warming level. **a**–**e**, Crop groups. Basemaps in **a**–**e** from Natural Earth (naturalearthdata.com).

Like for food crops in total, within crop groups, potential diversity decreases in the equatorial region and increases in other areas (Fig. 6, Extended Data Fig. 5 and Supplementary Figs. 1–3). Already under 2 °C warming, potential diversity would decrease on a larger share of cropland area than it would increase in all crop groups (Supplementary Table 5). Cereals and pulses would face the most spatially extensive decrease in potential diversity. For cereals, the share of cropland area where potential diversity decreases would range from 34% to 68% under 1.5 °C to 4 °C warming and for pulses, it would range from 33% to 57% (Supplementary Table 5). Notably, these two crop groups have been expected to offer considerable potential for climate change adaptation^22^ and improving food system sustainability^40^ through crop rotations. On the other hand, starchy roots and oil crops face the most severe decrease in potential diversity under all warming levels: starchy roots would lose all potential diversity (that is, −100%) on 12% of the cropland area already under 2 °C warming and oil crops on 11% (Supplementary Table 5). Moreover, oil crops have the largest shares of cropland area that would not be within the SCS under either baseline or future climate conditions: approximately 15% under all warming levels (Fig. 6 and Supplementary Table 5). This could imply that the climatic niche for oil crops is narrow compared with that of other crop groups. However, because the crop-specific SCSs were defined based on the climate conditions in the current production areas of each crop, part of the differences in results between crop groups can be attributed to differences in the geographical extent of the current cultivation areas (Supplementary Fig. 1 and Supplementary Note 1).

We also identified the croplands with emerging climatic potential for each crop group on the extent of the current total cropland. For crop groups, the cropland with emerging climatic potential currently hosts marginal or no production of crops within the crop group but would shift into the SCS of at least one crop within the group in a future projection. Regional summarization shows that these areas are concentrated in Europe and Central Asia, North America and the Middle East and North Africa (Supplementary Table 6).

## Discussion

Our analysis shows that in the equatorial region, even under 1.5 °C to 2 °C global warming, there are croplands where the climate will shift to conditions where none of the 30 studied crop types are currently grown. The resulting risk to current crop production adds further pressure to the already insufficient local food supply and threatens the livelihoods of agricultural households in several countries in this region^41–43^. The study covers four global warming levels ranging from 1.5 °C to 4 °C, and we find a considerable increase in the share of agricultural areas shifting to unprecedented climatic conditions if global warming exceeds 2 °C. This finding echoes existing research^7,17^ and the Paris agreement^44^, emphasizing the need to limit global warming to 2 °C to avoid detrimental impacts on food crop production, especially at low latitudes.

Furthermore, our study provides a comprehensive view of the effects of climate change on a wide and diverse range of food crops using a unified framework. This extends existing knowledge, which is concentrated on a limited number of crops^17,24,25,31–33,45^ and obtained using a wide variety of methods and climate change scenarios^33,46,47^. Hence, our approach allows a quantitative and comprehensive comparison of climate change impacts across crops and regions. The climate suitability estimates produced for food crops are in line with other more complex suitability maps, such as Global Agro-Ecological Zones v4 (GAEZ v4)^32^, Manners and Van Etten^45^, Cronin et al.^48^, Zabel et al.^49^ and Gardner et al.^12^, especially under 1.5 °C and 2 °C warming. Under the higher warming levels, our estimates show more adverse development, which might be due to the more rapid warming projected by the Coupled Model Intercomparison Project phase 6 (CMIP6) models used in this study compared with the CMIP5 and former models^34,50^ used in the other suitability maps. Alarmingly, we find that the largest adverse effects on current crop production are observed for crops and crop groups that are important elements of the food supply in their current major production areas^41,51^ (Fig. 2 and Extended Data Fig. 4). This effect is especially pronounced for tropical roots and cereals, suggesting a need for dietary shifts or for increasing food imports. Furthermore, we show that the four global staple crops (wheat, rice, maize and soybean) face some of the largest reductions in cropland area within the SCS, which underlines the need for diversifying crop production^13,17,19,22^.

Existing research has shown that climate change will make climate conditions more challenging for food crop production, especially at low latitudes, but make them more favourable for some crops in other areas^3,49,52–55^. Our research goes beyond this by identifying a similar global pattern for the future potential diversity of food crops (Figs. 5 and 6 and Supplementary Figs. 2 and 3). We find substantial increases in potential crop diversity in mid- to high-latitude regions, especially in North America, providing emerging climatic potential for transformational climate change adaptation and improving the climate resilience of crop production through diversifying production^13,17,19,22^ or selecting climate-change-resilient crops^2,13,21^. On the other hand, the largest and most severe reductions in potential crop diversity (that is, potential diversity given temperature and moisture conditions) are concentrated in the equatorial region and sub-Saharan Africa, which also have the lowest adaptive capacity worldwide^56^, further increasing the vulnerability of local crop production and livelihoods to the rapidly changing climate conditions^43^. With nearly 40% of cropland area shifting beyond the SCS of all the 30 analysed major food crop types in sub-Saharan Africa under 3 °C global warming, together with the projected rapid population growth in the twenty-first century (ref. ^57^), the effects on local food security could be drastic. It is unlikely that the adverse effects on crop production in low-latitude regions could be offset by incremental adaptations in agricultural management practices^17^. Therefore, in addition to climate change mitigation efforts, it is critical to support the food supply in these regions by strengthening national and international climate governance, for example, by creating trade arrangements and financing for innovative adaptation in low-income countries^17,58^.

Our analysis is based on the SCS concept^7^, and we define the climatic niche for each crop based on the current climate conditions in their current production areas. This means that when cropland shifts outside of the SCS, globally, there is no reference of, for example, agricultural management practices that would support continuing crop production under these novel climate conditions. Although the effect of changing climate in such areas could be mitigated through, for example, adopting improved management practices^59^ or new crop varieties^60–62^, these adaptations require considerable investment^59^ and are less accessible for farmers in developing countries^60^. On the other hand, we recognize that on croplands shifting into the SCS of locally new crops, there might be various socio-economic, environmental, phenological or management-related hindrances for adopting their cultivation, for example, market incentives^10,11^, cultural acceptance of diet change^63^, climate variability within the year^64,65^, length of growing season^64^, availability of irrigation and agricultural inputs^65–67^ and adaptation of humans to increasing temperatures^68^. These elements of suitability should be considered in future research to better understand the optimal crops locally under a changing climate. Additionally, this kind of optimization analysis would need to consider, for example, soil characteristics^69^, the effect of CO_2_ fertilization on crop yields^70^, and the competition of cropland between food, feed and biofuel crops, reforestation and other land uses for climate change mitigation^17^.

To conclude, there is a critical need to alleviate the negative impacts of climate change on food crop production and future potential crop diversity. The results should be linked to the wider context of climate change adaptation, including, for example, selecting climate-resilient crop varieties^60^ and optimizing management practices such as sowing dates, irrigation and fertilization^71^ and mitigation arising in the agricultural sector, for example, through improved and more sustainable cropland management and diet change^72^. Future research and solutions should aim at advancing the practical implementation of climate change mitigation and adaptation and addressing the current barriers to these actions (for example, low adaptive capacity, uncoordinated national policies and trade restrictions^17,73^).

## Methods

### Data

For all analyses, we utilized openly available, global gridded datasets. We used current (that is, ‘baseline’) and future estimates of precipitation and temperature from WorldClim2.1^36^ to delineate crop-specific Safe Climatic Spaces (SCSs) and to project future changes in cultivation areas within the SCS. Specifically, we obtained monthly precipitation and monthly minimum and maximum temperature data for every year in the baseline period (1990–2020) and 20-year averages of monthly precipitation and monthly minimum and maximum temperatures for future periods and Shared Socio-economic Pathways (SSPs) as specified in Table 1. For the future projections, we included data from eight General Circulation Models (GCMs) from the CMIP6. The included CMIP6 models were BCC-CSM2-MR, CNRM-CM6-1, CNRM-ESM2-1, canesm5, IPSL-CM6A-LR, MIROC-ES2L, MIROC6 and MRI-ESM2-0. Additionally, we obtained 30-year averages of the monthly minimum, maximum and average temperatures from WorldClim2.1^36^ for the historical period 1970–2000 for temperature bias correction. All climate data have a 5-arcmin resolution.

To determine the volume and geographical extent of the current crop production in the main analyses, we used the SPAM 2020^35^ crop production and physical cropland area data. The results were validated using SPAM 2010^38^ and SPAM 2005^37^ crop production and physical cropland area data (Supplementary Note 1). The datasets cover all crop types reported by the Food and Agriculture Organization of the United Nations, except for crops that are exclusively used as fodder, for example, grasses^74^. The data have 5-arcminute resolution and represent production in metric tons and physical cropland area in hectares in 2020, 2010 and 2005. These datasets aggregate production from different farming systems: irrigated high-input and rainfed high-input, rainfed low-input and rainfed subsistence production. The SPAM 2020 data include 46 crop types, 30 of which we classified as food crops following the SPAM 2010 data description (Table S3 in Yu et al.)^74^ (Supplementary Table 1). In addition, we classified tomato, onion and citrus fruits, which are found only in the SPAM 2020 data, as food crops. The SPAM 2005 and SPAM 2010 datasets include 42 crop types, 27 of which we classified as food crops following the SPAM 2010 data description^74^. We grouped the food crops into five crop groups for some visualizations, following the categorization in the SPAM 2010 data description^74^ and categorized tomato, onion and citrus fruits as ‘fruits and vegetables’ (Supplementary Table 1). We used the physical area data of all 46 (SPAM 2020, main analysis) and 42 (SPAM 2005 and SPAM 2010; Supplementary Note 1) crops to calculate the global and regional physical areas of croplands and to create total cropland masks for the matching crop data years. In contrast to the data specifications (https://mapspam.info/methodology/), the combined physical cropland area of all crops in a grid cell exceeded the physical area of the grid cell for approximately 6% of the cropland area in the SPAM 2020 data and for approximately 5% of the cropland area in the SPAM 2005 data. These inconsistencies are mainly located in Nigeria and India in the SPAM 2020 data and in India in the SPAM 2005 data. Because of the minor area, no changes were made due to this.

To produce regionally aggregated results, we used the World Bank regional division^75^. The regions are East Asia and the Pacific, Europe and Central Asia, Latin America and the Caribbean, the Middle East and North Africa, North America, South Asia and sub-Saharan Africa (Supplementary Fig. 4).

### SCS concept

The SCS concept^7^ defines the climatic conditions that support the highest 95% of the current food crop production. This is analogous to the climatic niche concept, which is commonly used for modelling the effects of climate change on future crop species distributions^8,26,31,62,76^. Our analysis builds on the evidence that although the climatic niches of crops may shift^76^, the climate changes faster than the niches have historically been able to shift^8^. The SCS concept utilizes the Holdridge Life Zone (HLZ) framework^77^, which divides the Earth into 38 climate zones based on annual precipitation, biotemperature (mean of monthly average temperatures above 0 °C (ref. ^7^)) and aridity (the ratio between average annual potential evapotranspiration (PET) and precipitation). We applied the SCS concept and the methodology in Kummu et al.^7^ to delineate climatically fixed, crop-specific SCSs using climate data from 1990–2020 (Extended Data Fig. 1). In an additional analysis for maize and soybean, we defined the SCS for the cropping season only (Supplementary Note 1). Only these two crops were selected for the seasonal analysis because of the limited spatial coverage of crop calendar data for other crops^78^ matching SPAM crop types. We extended the analyses to the total cropland area of the 46 crop types in SPAM 2020^35^ (or 42 in SPAM 2010^38^ and SPAM 2005^37^; Supplementary Note 1), which allows analysis of the future changes in the potential diversity of food crops regardless of their current cultivation areas (Extended Data Fig. 2).

Compared with other, more comprehensive methods of assessing climate or land suitability for food crops^32,48^, the SCS methodology requires less data, which makes it more feasible to apply both globally and in data-scarce regions for many crops and for a variety of climate change scenarios. Moreover, because we project future shifts in areas within the SCS on the current total cropland, we can assume that some suitability factors beyond climate conditions are satisfied, for example, terrain suitability and land availability for agricultural use and that these conditions change at a slower rate than the climate currently. However, there are suitability factors that are not considered in the analysis, such as consumer and producer acceptance of each crop beyond their current cultivation areas^63^. Moreover, it must be noted that the SCS methodology defines the climatic niche for each crop as the climate conditions where the current agricultural management practices (including irrigation and agricultural inputs) can support major food crop production and does not consider potential future changes in management practices. Thus, areas outside the SCS could still support crop cultivation with new practices, whereas areas within the SCS might face challenges due to changes, for example, in availability of agricultural inputs.

### Climate parameter calculation

#### Holdridge life zone parameters

The climate parameters that are needed for Holdridge life zone classification and for defining crop-specific SCSs (annual precipitation, biotemperature and aridity) and a frost threshold parameter were derived from the WorldClim2.1^36^ data for the baseline period 1990–2020 and for the four future periods following the corresponding SSPs (Data), applying the methodology in Kummu et al.^7^ and Holdridge^77^. For the baseline period, we obtained the average annual precipitation by first calculating monthly averages of precipitation over the baseline years and then calculating the sum of the monthly averages. For all future periods, we obtained the average annual precipitation by calculating the sum of the 20-year average monthly precipitation from the WorldClim2.1 data. The biotemperature was calculated from the average monthly temperature. Because that was not available for the whole baseline period 1990–2020 or for future periods, we estimated the average temperature as the average of the monthly minimum and maximum temperatures. For the baseline period, those were calculated by taking the monthly averages of the minimum and maximum temperatures. For the future period, we directly used the 20-year monthly minimum and maximum temperatures available in WorldClim2.1. The monthly average estimation was bias corrected using the monthly average, minimum and maximum temperatures from the historical period of 1970–2000. To calculate the annual biotemperature, we took the average of the average monthly temperatures above 0 °C. The aridity parameter was calculated as the annual PET divided by the annual average precipitation. The annual PET was obtained by multiplying the average monthly biotemperature by the constant 58.93, as defined in Holdridge^77^ and summing the monthly values. Finally, we calculated a binary, annual frost threshold parameter to delineate areas with no subzero temperatures. The parameter was used to separate between temperate and subtropical zones^7^ and it was calculated for all periods using monthly minimum temperatures. The baseline monthly minimum temperatures were averaged from data over individual years in the baseline period, and the future monthly minimums were obtained directly from the WorldClim2.1 data.

#### Global warming levels

We show all results for four global warming levels (1.5 °C, 2 °C, 3 °C and 4 °C), aiming to relate them to international targets such as the Paris agreement^44^ and IPCC reports. The Holdridge life zone climate parameter data to represent climate conditions under each warming level were chosen based on IPCC central estimates of years when each warming level is reached, following the different SSPs^34^. The warming levels are temperature anomalies from the pre-industrial period (1850–1900), and the estimates of timing integrate unaltered CMIP6 multimodel projections that incorporate constraints from warming in the past decades and likely ranges of the transient climate response and the equilibrium climate sensitivity of CMIP6 models^34^. The warming levels may be represented by data from different time periods and SSPs because comparisons of projections from CMIP6 models for each warming level have shown that regional, seasonal and annual patterns in both mean temperature and precipitation respond to specific global warming similarly regardless of the time at which the specific warming level is reached^34^.

To calculate the HLZ parameter data to represent each warming level, we selected the SSP that first crosses the warming level in the IPCC estimates^34^ and the previously calculated HLZ parameter data from the two WorldClim2.1^36^ data future periods that overlap with the IPCC central estimate year range (Table 1). To obtain data on each HLZ parameter to represent the IPCC estimate year ranges, we linearly interpolated between HLZ parameter data from the overlapping WorldClim data year ranges at the grid cell level as follows:

1 $$Y=\,\frac{{Y}_{0}({x}_{1}-x)+\,{Y}_{1}(x-{x}_{0})\,}{({x}_{1}-{x}_{0})}\,$$

where ${Y}_{0}$ is the HLZ parameter data from the earlier WorldClim data year range, ${Y}_{1}$ is the HLZ parameter data from the later year range, ${x}_{0}$ is the earlier WorldClim data year range centre year, ${x}_{1}$ is the later centre year and $x$ is the IPCC central estimate centre year (Table 1). For example, the HLZ data representing the warming level 1.5 °C were interpolated from WorldClim data for 2021–2040 and 2041–2060, representing SSP1–2.6, with WorldClim centre years ${x}_{0}$ = 2031 and ${x}_{1}$ = 2051 and IPCC centre year *x* = 2033.

### Crop-specific SCSs and projecting their future geographic shifts

We delineated the crop-specific SCSs based on the current (1990*–*2020) climatic extent of the major production areas of each of the 30 food crops in 2020 by applying the method in Kummu et al.^7^ for individual food crops. We used the highest 95% of the production of each crop (in metric tons) as the threshold for major production, indicating that these production areas are within the climatic niche of that crop. Furthermore, by using the 95% threshold, we excluded marginally suitable climate conditions from the SCS. Using wheat as an example, the current climatic extent of wheat production was defined by mapping each grid cell that currently hosts major wheat production to the three climate parameters: precipitation, biotemperature and aridity (Extended Data Fig. 1). To examine the impacts of climate change on the current wheat production, we compared the future climate conditions of the current wheat production areas to the SCS of wheat (Extended Data Fig. 1).

For projections extending to the current total cropland, that is, beyond the current cultivation areas of individual crops but within that of all crops, we mapped each grid cell of the total cropland area on the three climate parameters. Then, we examined whether the cropland areas fell within or outside the crop-specific SCSs (Extended Data Figs. 1 and 2). The projections on the total cropland were performed both in the baseline and future climate conditions under the four global warming levels to analyse potential crop diversity beyond the current cultivation areas of each crop (but never exceeding the current total cropland of all crops). Whether a grid cell on the cropland was within the SCS of a crop was determined either based on the majority vote of the eight GCMs (Figs. 1, 5 and 6) or based on the median of estimations from the GCMs (Figs. 2, 3 and 4).

### Climate risk to current crop production

We analysed the future climate risk to current crop production by two indicators: (1) finding the lowest global warming level that would push 25%, 50% and 75% of the current crop production in each location outside of the crop-specific SCS and (2) for each crop and crop group, calculating the total share of current production that would fall outside the crop-specific SCS under warming levels from 1.5 °C to 4 °C, aggregated regionally and globally.

For the first risk indicator, we initially calculated the share of the total current production of the 30 food crops that would fall outside of crop-specific SCSs in each grid cell under the four global warming levels. For each crop and global warming level, we determined a location outside the SCS of a crop if at least half of the eight GCMs estimated that the location was outside the crop-specific SCS. We then multiplied these crop-specific estimates by the current production of each crop, extracted the total, grid cell-level production volume that would be outside of the SCS under each global warming level and transformed that value into the share of total production. Finally, we compared the grid-cell-level share of production outside the SCS under each warming level to the percentage thresholds (25%, 50% and 75%) to find the lowest global warming level where the percentage threshold of production in risk would be exceeded. The grid cell-level results using the 25% threshold were summarized within geographical regions as the share of regional cropland area shifting to risk under each warming level.

For the second risk indicator, we utilized all eight GCM estimates of the volume of the current production of each crop that would fall outside the crop-specific SCS. This was determined by finding the current production areas (grid cells) that the GCMs estimated outside the SCS of each crop and summarizing the production from those areas. We performed the analysis for individual crops at the global scale and for crop groups and all crops in total at the global and regional scales. For individual crops, we calculated the median and the 25th and 75th percentiles of GCM estimates of crop-specific production outside the SCS (globally and within regions) and divided that by the total reference production of the crop (globally and within regions). Within crop groups and all crops in total, we summarized the individual crop medians and the 25th and 75th percentiles of production outside the SCS and divided those by the sum of the reference production within the group or the reference production of all crops.

### Change in total cropland area within crop-specific SCSs

Similarly, we determined the change in the total cropland area within the SCSs of individual crops and crop groups. The analysis of locations within the SCS was performed at the grid cell level, and these areas were subsequently summarized globally. The analysis was performed under baseline climate conditions and under the four global warming levels and extended to the total cropland area of all crops, regardless of the current cultivation areas of individual crops (Extended Data Fig. 2). Again, for the future projections, we utilized estimates of total cropland area within the SCS from the eight GCMs and calculated their median as well as the 25th and 75th percentiles. For individual crops and for crop groups, we calculated the GCM median and the 25th and 75th percentiles of percentage net change in total cropland area within the SCS. In addition, for crop groups, we determined the GCM median as well as the 25th and 75th percentiles of the ratios of gained and lost cropland area within the SCS to the baseline cropland area within the SCS. The percentage net changes were defined as follows:

2 $$\frac{{A}_\mathrm{warming}-\,{A}_\mathrm{baseline}}{{A}_\mathrm{baseline}}\,\times\,100 \%$$

where ${A}_\mathrm{warming}$ is the total cropland area within the SCS of a crop under a warming level. ${A}_\mathrm{baseline}$ is the total cropland area within the SCS of a crop in the baseline climate.

The ratio of gained and lost cropland area within the SCS to the baseline cropland area within the SCS was defined as follows:

3 $$\frac{{A}_\mathrm{change}}{{A}_\mathrm{baseline}}\,\times\,100 \%$$

where ${A}_\mathrm{change}$ is the gained suitable cropland area (that is, the cropland area that shifts within the SCS under a warming level) or the lost suitable cropland area (that is, the cropland area that shifts outside the SCS under a warming level). ${A}_\mathrm{baseline}$ is the total cropland area within the SCS of a crop in the baseline climate.

### Projected changes in potential crop diversity

We examined changes in the global potential crop diversity utilizing the crop-specific total cropland within the SCSs. Here the potential diversity in a location (grid cell) refers to the number of crops whose SCSs match the local climate conditions. We use the term ‘potential’ because we examine the climatic suitability beyond the current cultivation areas of individual crops. We determined the total potential crop diversity for all 30 food crops and the five crop groups. The change from the baseline was calculated as percentage change in the number of possible crops in future conditions compared with the number in baseline conditions. Furthermore, we identified the locations where there is currently marginal or no crop (or crop group) production but where the climate conditions would shift into the SCS of at least one crop in the future (that is, ‘cropland with emerging climatic potential’) and the locations that are not climatically within the SCS of any crop under either baseline or future climate conditions. At the level of a crop group, for example, cereals, cropland with emerging climatic potential is currently not climatically within the SCS of any cereal crop but would shift within the SCS of at least one cereal crop in the future. The grid-cell-level estimates of changes in potential diversity were also summarized within geographical regions and latitude and elevation zones^79^ (Supplementary Note 2) as share of regional cropland area in categories of percentage change (that is, −100%, −99.99 to −75%, −75% to −50% and so on).

### Reporting summary

Further information on research design is available in the Nature Portfolio Reporting Summary linked to this article.

## Supplementary information

Supplementary Information Supplementary Tables 1–16, Figs. 1–6 and Notes 1 and 2.

Reporting Summary

Supplementary Data 1 Source data for Supplementary Fig. 6.

## Source data

Source Data Fig. 1 Source data for bar plot in **b**.

Source Data Fig. 2 Source data for bar plots and their elements in **a**–**g**.

Source Data Fig. 3 Source data for bar plot and its elements.

Source Data Fig. 4 Source data for bar plot.

Source Data Fig. 5 Source data for all bar plots.

Source Data Extended Data Fig. 1 Source data for scatter points.

Source Data Extended Data Fig. 4 Source data for bar plots and their elements in **a**–**f**.

## Data Availability

The climate parameter data were obtained from WorldClim2 (https://www.worldclim.org/data/index.html) for historical periods 1970–2000 (30-year averages of monthly minimum, maximum and mean temperatures and precipitation) and 1990–2020 (monthly minimum and maximum temperatures and precipitation) and for the following future period SSPs and GCMs: SSP1–2.6 periods 2021–2040 and 2041–2060; SSP2–4.5 periods 2041–2060 and 2061–2080; SSP3–7.0 periods 2061–2080 and 2081–2100; and SSP5–8.5 periods 2061–2080 and 2081–2100 (20-year averages of monthly minimum and maximum temperature and precipitation), all for the GCMs BCC-CSM2-MR, CNRM-CM6-1, CNRM-ESM2-1, canesm5, IPSL-CM6A-LR, MIROC-ES2L, MIROC6 and MRI-ESM2-0. Crop-specific production data (metric tons) and physical cropland areas (hectares) were obtained from SPAM 2020 v1.0 (https://dataverse.harvard.edu/dataset.xhtml?persistentId=doi:10.7910/DVN/SWPENT#), SPAM 2010 v2.0 (https://dataverse.harvard.edu/dataset.xhtml?persistentId=doi:10.7910/DVN/PRFF8V#) and SPAM 2005 v3.2 (https://dataverse.harvard.edu/dataset.xhtml?persistentId=doi:10.7910/DVN/DHXBJX#). Crop calendars (sowing date and harvest date) for maize and soybean, both rainfed and irrigated, were obtained from the GGCMI Phase 3 crop calendar via Zenodo at 10.5281/zenodo.5062513 (ref. ^78^). The void-filled Digital Elevation Model was obtained from HydroSHEDS v1 (https://data.hydrosheds.org/file/hydrosheds-v1-dem/hyd_glo_dem_30s.zip). The data produced in this study are openly available via Zenodo at 10.5281/zenodo.14801623 (ref. ^80^). These data include source data tables and images for all figures, Extended Data figures and Supplementary figures and raster files of other outputs generated in this study. Source data are provided with this paper. Code availability

## Notes

The data analysis was carried out in MATLAB and R. The code used to perform the analyses in this study is available via Zenodo at 10.5281/zenodo.14804349 (ref. ^81^).
