# Supplementary material for: Climate change threatens crop diversity at low latitudes
Source: Nat Food. 2025 Mar 4;6(4):331–42. doi: 10.1038/s43016-025-01135-w (PMC12018264; doi:10.1038/s43016-025-01135-w)
Supplement: Supplementary file 1 — Supplementary Tables 1–16, Figs. 1–6 and Notes 1 and 2. [file 43016_2025_1135_MOESM1_ESM.pdf]

nature food

Supplementary information https://doi.org/10.1038/s43016-025-01135-w

Climate change threatens crop diversity at low latitudes

In the format provided by the authors and unedited

Table of Contents

Supplementary tables 2
Supplementary figures 7
Supplementary Note 1 11
Uncertainty analysis with the SPAM 2005 and SPAM 2010 crop production datasets .. 11
Uncertainty analysis with Safe Climatic Space defined based on crop calendar 18
Supplementary Note 2 21
Changes in potential crop diversity within elevation and latitude zones 21
Supplementary references 23

Supplementary tables

Supplementary Table 1 Crop classification into crop groups following the SPAM 2010 data description. *Adapted from Table S3 in Yu et al[1]. Crops marked with an asterisk (*) are included only in the main analyses using SPAM 2020 crop production data. Other crops are included in both the main analyses and in the supplementary analyses using the SPAM 2010 and SPAM 2005 crop production datasets (see Supplementary Note 1).*

**Crop groups**	**Crops**
Cereals	Wheat, rice, maize, barley, pearl millet, small millet, sorghum, other cereals
Starchy roots	Potato, sweet potato, yams, cassava, other roots
Pulses	Bean, chickpea, cowpea, pigeon pea, lentil, other pulses
Oil crops	Soybean, ground nut, coconut
Fruits and vegetables	Banana, plantain, tropical fruit, temperate fruit, citrus fruit*, tomato*, onion*, other vegetables.

Supplementary Table 2 Cumulative share of cropland area where current crop production would be pushed into considerable risk under global warming levels. *Considerable risk is defined as least 25% [50%, 75%] of the current production of the 30 analysed food crops shifting outside the crop-specific Safe Climatic Spaces (SCS). "Cropland within SCS under all warming levels" shows the share of cropland area where less than 25% [50%, 75%] of current production would be pushed outside the crop-specific SCS under all warming levels.*

	**Cumulative share of cropland area where at least 25% of production shifts outside the SCS under warming level (%)**				**Cropland within SCS under all warming levels (%)**
**Region**	**1.5°C**	**2°C**	**3°C**	**4°C**	
Global	15 [10, 7]	23 [17, 13]	41 [34, 29]	55 [49, 44]	45 [51, 56]
East Asia & Pacific	13 [9, 8]	23 [17, 15]	41 [35, 32]	51 [44, 41]	49 [56, 59]
Europe & Central Asia	8 [6, 5]	9 [7, 5]	13 [10, 8]	23 [16, 13]	77 [84, 87]
Latin America & Caribbean	11 [7, 5]	16 [11, 8]	32 [26, 21]	52 [45, 40]	48 [55, 60]
Middle East & North Africa	46 [35, 26]	55 [44, 33]	69 [59, 48]	79 [72, 63]	21 [28, 37]
North America	11 [5, 4]	12 [6, 5]	16 [9, 6]	20 [12, 9]	80 [88, 91]
South Asia	16 [11, 7]	28 [19, 14]	60 [47, 37]	89 [81, 71]	11 [19, 29]
Sub-Saharan Africa	21 [12, 7]	37 [27, 20]	66 [60, 56]	78 [74, 71]	22 [26, 29]

Supplementary Table 3 Percentage net change in cropland area within the crop-specific Safe Climatic Space (SCS) of 30 food crops under global warming levels. *The percentage change was calculated as the change in area within the SCS at the warming level compared to the cropland area within the SCS under baseline climate. 'Med' indicates the median estimate from 8 General Circulation Models (GCM), and '25th' and '75th' indicate the 25th and 75th percentiles of the GCM estimates, respectively.*

	**Global warming (°C), measurement**			
**Crop**	**1.5, med [25th, 75th]**	**2, med [25th, 75th]**	**3, med [25th, 75th]**	**4, med [25th, 75th]**
Wheat	-9 [-11, -8]	-15 [-19, -14]	-28 [-31, -25]	-38 [-42, -35]
Rice	-3 [-3, -2]	-7 [-11, -6]	-22 [-28, -18]	-40 [-51, -33]
Maize	-1 [-3, -1]	-7 [-11, -4]	-23 [-27, -17]	-37 [-44, -33]
Barley	-8 [-9, -7]	-13 [-14, -11]	-22 [-23, -19]	-30 [-33, -27]
Small millet	-1 [-1, -1]	-4 [-6, -3]	-14 [-17, -10]	-30 [-40, -24]
Peal millet	2 [1, 4]	1 [0, 3]	-16 [-22, -10]	-39 [-51, -31]
Sorghum	3 [3, 4]	2 [-1, 4]	-10 [-13, -4]	-23 [-32, -17]
Other cereals	-3 [-3, -2]	-7 [-9, -5]	-20 [-24, -14]	-34 [-42, -31]
Potato	-9 [-10, -7]	-14 [-18, -13]	-28 [-31, -24]	-40 [-45, -36]
Sweet potato	0 [-3, 1]	-6 [-12, -3]	-23 [-29, -18]	-39 [-43, -32]
Yams	-1 [-2, 0]	-8 [-15, -5]	-34 [-41, -28]	-60 [-67, -51]
Cassava	0 [-1, 1]	-6 [-10, -3]	-28 [-32, -22]	-44 [-50, -36]
Other roots	-4 [-6, -2]	-10 [-16, -7]	-28 [-33, -22]	-42 [-46, -35]
Bean	-1 [-1, 0]	-3 [-5, -2]	-16 [-21, -12]	-31 [-39, -25]
Chickpea	-2 [-3, -1]	-6 [-7, -3]	-18 [-21, -14]	-33 [-35, -27]
Cowpea	2 [2, 3]	-1 [-4, 3]	-22 [-30, -15]	-46 [-54, -36]
Pigeon pea	-3 [-4, -1]	-9 [-14, -6]	-31 [-36, -25]	-50 [-51, -43]
Lentil	-11 [-14, -10]	-18 [-22, -16]	-31 [-32, -28]	-39 [-43, -37]
Other pulses	-6 [-7, -5]	-12 [-15, -10]	-26 [-30, -22]	-38 [-44, -35]
Soybean	-8 [-10, -6]	-16 [-20, -14]	-35 [-41, -28]	-49 [-52, -46]
Groundnut	4 [3, 5]	1 [-1, 4]	-14 [-18, -8]	-31 [-38, -22]
Coconut	6 [5, 7]	0 [-4, 3]	-26 [-32, -18]	-50 [-59, -39]
Banana	5 [4, 5]	3 [1, 6]	-13 [-18, -6]	-29 [-37, -19]
Plantain	1 [0, 2]	-6 [-12, -3]	-31 [-35, -24]	-47 [-49, -38]
Citrus fruit	-3 [-5, -1]	-8 [-14, -6]	-23 [-27, -18]	-30 [-35, -28]
Tropical fruit	2 [2, 3]	0 [-3, 3]	-12 [-15, -7]	-26 [-33, -19]
Temperate fruit	-1 [-2, 0]	-5 [-8, -3]	-20 [-24, -15]	-34 [-41, -29]
Tomato	1 [0, 1]	-3 [-6, -1]	-16 [-20, -12]	-29 [-35, -24]
Onion	1 [0, 2]	-3 [-6, -1]	-17 [-20, -13]	-30 [-37, -25]
Other vegetables	0 [-1, 0]	-5 [-9, -4]	-20 [-24, -16]	-34 [-42, -29]

Supplementary Table 4 Global share of cropland in categories of change in potential crop diversity under global warming. *The change in potential diversity of the 30 analysed food crops was calculated by comparing the potential diversity at the warming level to baseline potential diversity in 1990–2020 climate. "Cropland with emerging climatic potential" indicates that the area hosts marginal crop production in the baseline climate but would shift into the Safe Climatic Space (SCS) of at least one crop under the warming level. "Marginal in baseline and outside the SCS under warming level" indicates that the area hosts marginal production in the baseline climate and is not within the SCS of any crop under the warming level.*

	**Share of global cropland in category under global warming levels (%)**			
**Potential diversity change compared to baseline (%)**	**1.5°C**	**2°C**	**3°C**	**4°C**
-100	1.3	3.9	15.8	29.7
-99.99 to -75	1.6	3.5	7.0	7.1
-75 to -50	3.6	6.3	6.4	6.1
-50 to -25	8.0	10.1	7.9	6.4
-25 to 0	28.7	28.3	19.1	12.2
no change	22.9	13.0	11.7	9.8
0 to +25	24.1	23.8	18.4	14.9
+25 to +50	5.7	6.5	7.8	6.4
+50 to +75	1.9	2.3	3.3	3.8
+75 to +100	1.6	1.7	1.9	2.8
Cropland with emerging climatic potential	0.3	0.3	0.4	0.4
Marginal in baseline and outside the SCS under warming level	0.4	0.3	0.3	0.3

Supplementary Table 5 Global share of cropland in categories of change in potential crop diversity within crop groups. *The change in potential diversity of the crops in a crop group was calculated by comparing the potential crop group level diversity at the warming level to baseline potential crop group level diversity in 1990–2020 climate. Abbreviations: C: cereals, F: fruits and vegetables, O: oil crops, P: pulses, and S: starchy roots. "Cropland with emerging climatic potential" indicates that the area hosts marginal or no production of the crops in the group in the baseline climate but would shift into the Safe Climatic Space (SCS) of at least one crop in the group under the warming level. "Marginal in baseline and, outside the SCS under warming level" indicates that the area hosts marginal or no production of crops in the group in the baseline climate and is not within the SCS of any crop under the warming level.*

**Potential diversity change compared to baseline (%)**	**Share of global cropland in category under global warming level (%)**																			
	**1.5°C**					**2°C**					**3°C**					**4°C**				
	**C**	**F**	**O**	**P**	**S**	**C**	**F**	**O**	**P**	**S**	**C**	**F**	**O**	**P**	**S**	**C**	**F**	**O**	**P**	**S**
-100	2.4	2.6	5.9	3.0	6.3	6.2	5.3	10.5	6.6	12.2	19.4	17.2	23.9	19.9	26.0	32.5	31.5	37.4	33.6	38.4
-99.99 to -75	0.7	1.8	0.0	1.8	0.9	1.2	4.3	0.0	3.9	1.5	2.1	6.7	0.0	5.8	0.9	2.7	6.2	0.0	6.2	0.4
-75 to -50	4.8	3.3	4.7	10.8	5.3	7.5	4.7	6.9	12.7	5.9	8.3	4.1	7.5	11.8	5.3	10.0	3.3	7.4	8.1	4.9
-50 to -25	11.5	4.5	4.8	12.3	4.5	15.3	5.2	6.9	12.5	4.3	14.9	4.9	6.8	9.5	2.9	14.0	3.2	2.5	7.7	1.7
-25 to 0	14.9	7.5	0.0	4.7	2.2	16.0	6.6	0.0	7.2	3.0	12.7	3.6	0.0	5.6	1.9	9.1	2.5	0.0	0.9	0.2
no change	41.1	56.7	56.7	44.0	58.4	28.3	47.4	45.5	31.4	47.4	19.9	32.6	28.0	22.5	33.5	12.2	19.6	18.4	19.5	24.8
0 to +25	11.6	12.3	0.0	2.3	2.7	11.5	13.8	0.0	2.9	3.9	7.8	16.2	0.0	2.5	4.4	6.1	15.1	0.0	1.9	3.5
+25 to +50	6.7	3.5	3.6	9.3	4.4	6.3	4.7	4.6	10.2	5.3	6.5	6.7	6.2	9.4	6.0	4.9	9.8	5.6	8.8	6.0
+50 to +75	2.0	0.8	0.0	0.3	0.8	2.9	0.9	0.0	0.6	1.2	3.1	1.3	0.0	0.7	1.8	2.9	1.4	0.0	1.0	0.8
+75 to +100	2.1	2.3	7.8	6.6	6.9	2.7	2.0	9.7	7.2	7.7	3.5	1.7	11.2	7.4	9.6	3.7	2.3	11.7	7.3	11.1
Cropland with emerging climatic potential	0.4	2.5	0.9	0.8	0.5	0.5	3.1	1.0	1.2	0.7	0.6	3.3	1.2	1.6	0.4	0.7	3.2	1.4	1.7	0.5
Marginal in baseline and outside the SCS under warming level	1.6	2.2	15.7	4.0	7.0	1.5	1.9	14.9	3.7	6.9	1.3	1.6	15.2	3.3	7.2	1.2	1.7	15.6	3.3	7.7

Supplementary Table 6 Regional share of cropland with emerging climatic potential (%) for crop groups on the current total cropland under global warming. *Cropland with emerging climatic potential indicates that the area hosts marginal or no production of crops in the group in the baseline climate but would shift into the Safe Climatic Space (SCS) of at least one crop in the group under the warming level. Abbreviations: C: cereals, F: fruits and vegetables, O: oil crops, P: pulses, and S: starchy roots.*

**Region**	**1.5°C**					**2°C**					**3°C**					**4°C**				
	**C**	**F**	**O**	**P**	**S**	**C**	**F**	**O**	**P**	**S**	**C**	**F**	**O**	**P**	**S**	**C**	**F**	**O**	**P**	**S**
Global	0.4	2.5	0.9	0.8	0.5	0.5	3.1	1.0	1.2	0.7	0.6	3.3	1.2	1.6	0.4	0.7	3.2	1.4	1.7	0.5
East Asia & Pacific	0.5	0.9	0.9	0.8	0.3	0.7	1.1	0.8	1.1	0.4	0.8	1.5	1.2	1.7	0.6	1.0	1.8	2.1	2.0	0.8
Europe & Central Asia	0.4	4.7	1.0	0.6	0.2	0.5	6.3	0.6	0.9	0.0	0.5	7.0	0.9	1.4	0.1	0.4	6.4	1.0	1.4	0.1
Latin America & Caribbean	0.2	0.4	1.0	0.4	0.7	0.4	0.5	1.3	0.6	0.8	0.5	0.6	1.4	0.9	0.9	0.6	0.6	1.4	1.0	1.3
Middle East & North Africa	2.9	3.5	0.4	2.2	0.3	3.4	4.1	0.3	3.6	0.1	4.3	4.9	0.4	7.4	0.1	6.0	4.3	0.8	7.5	0.0
North America	0.6	11.6	0.6	2.6	0.2	0.6	14.1	0.7	3.7	0.1	0.6	14.3	0.8	4.0	0.1	0.5	14.3	0.8	3.7	0.2
South Asia	0.2	0.4	0.9	0.6	1.3	0.2	0.4	1.2	0.7	2.3	0.2	0.3	1.0	1.0	0.9	0.2	0.3	0.8	1.0	0.4
Sub-Saharan Africa	0.1	0.2	1.2	0.2	0.5	0.1	0.2	1.3	0.3	0.6	0.2	0.2	1.6	0.5	0.2	0.2	0.2	1.9	0.5	0.2

Supplementary figures

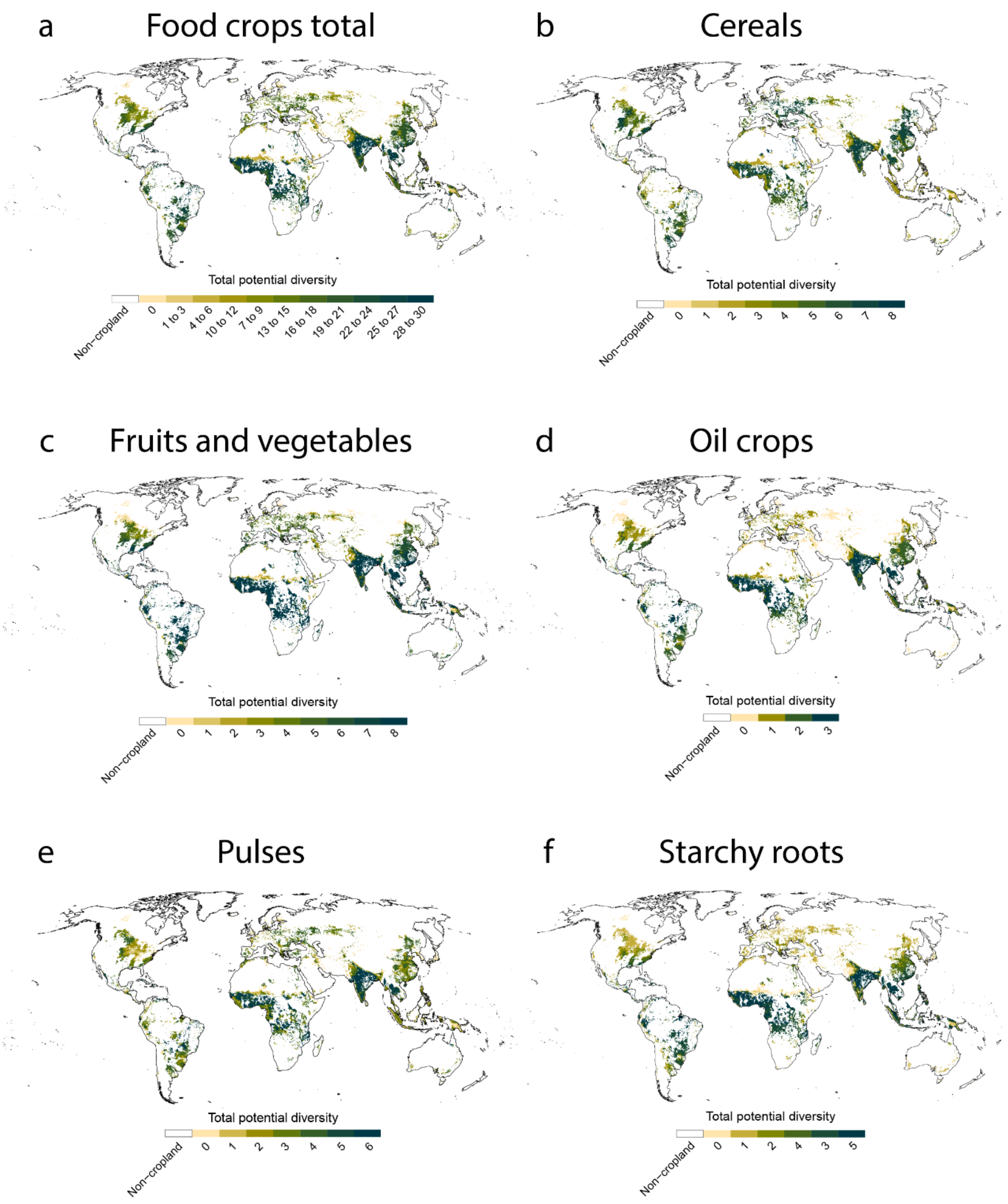

Supplementary Figure 1 Total potential food crop diversity under baseline climate conditions (1990–2020). *Potential crop diversity was determined by projecting the extent of cropland within the crop-specific Safe Climatic Spaces (SCS) of the 30 analysed food crops on the current total cropland, regardless of the current cultivation area of each crop. Potential diversity is measured as the number of crops that could be cultivated in each location (grid cell) based on whether the crop-specific SCS covers the location under the warming level. In each panel, the category showing the highest value indicates the maximum potential diversity in that crop group. Panel a): total number of food crops; panels b) – e): crop groups (Supplementary Table 1). Coastline is from Natural Earth (naturalearthdata.com).*

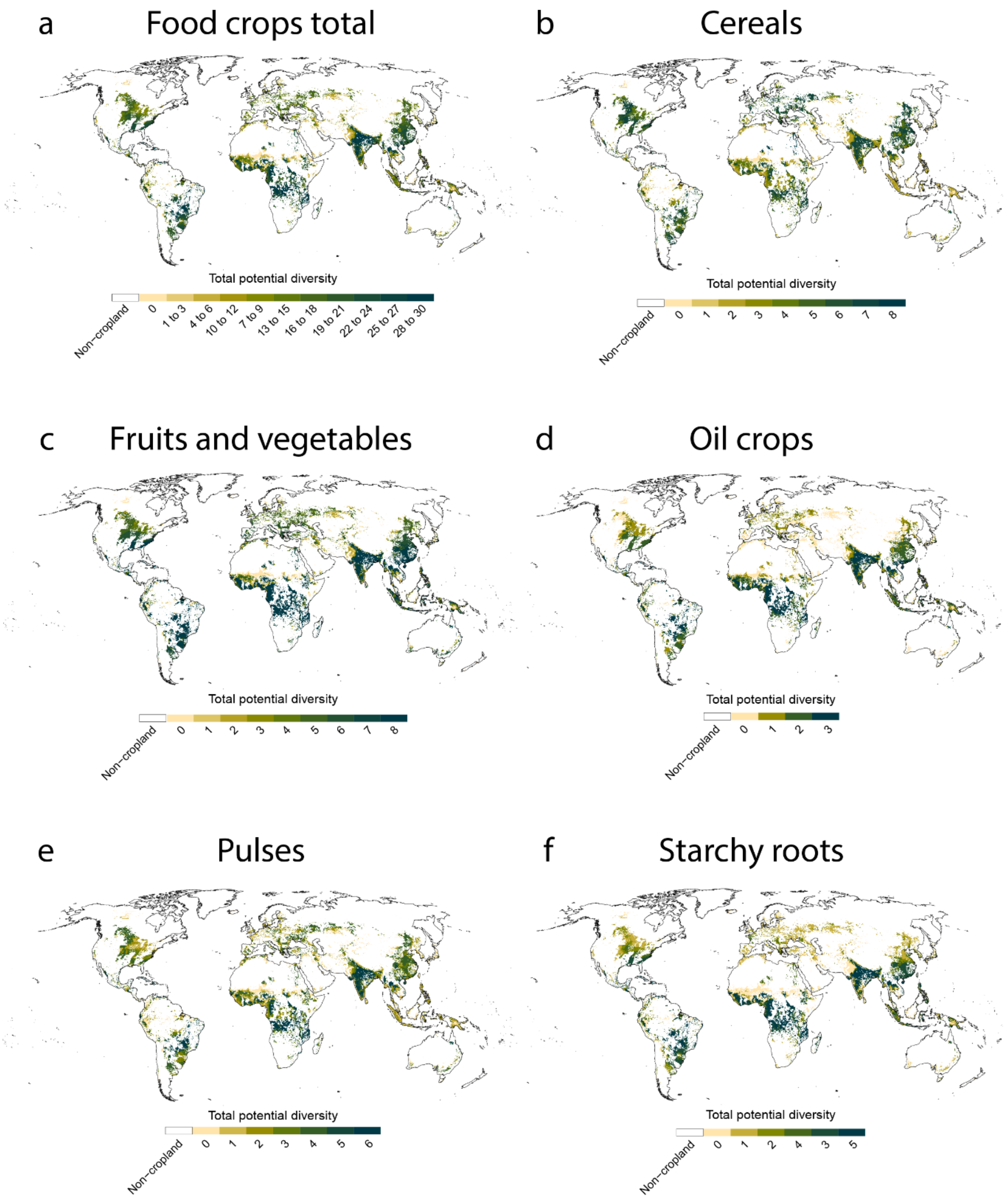

Supplementary Figure 2 Total potential food crop diversity under 2°C global warming. *Potential crop diversity was determined by projecting the extent of cropland within the crop-specific Safe Climatic Spaces (SCS) of the 30 analysed food crops on the current total cropland, regardless of the current cultivation area of each crop. Potential diversity is measured as the number of crops that could be cultivated in each location (grid cell) based on whether the crop-specific SCS covers the location under the warming level. In each panel, the category showing the highest value indicates the maximum potential diversity in that crop group. Panel a): food crops total; panels b) – e) crop groups (Supplementary Table 1). Coastline is from Natural Earth (naturalearthdata.com).*

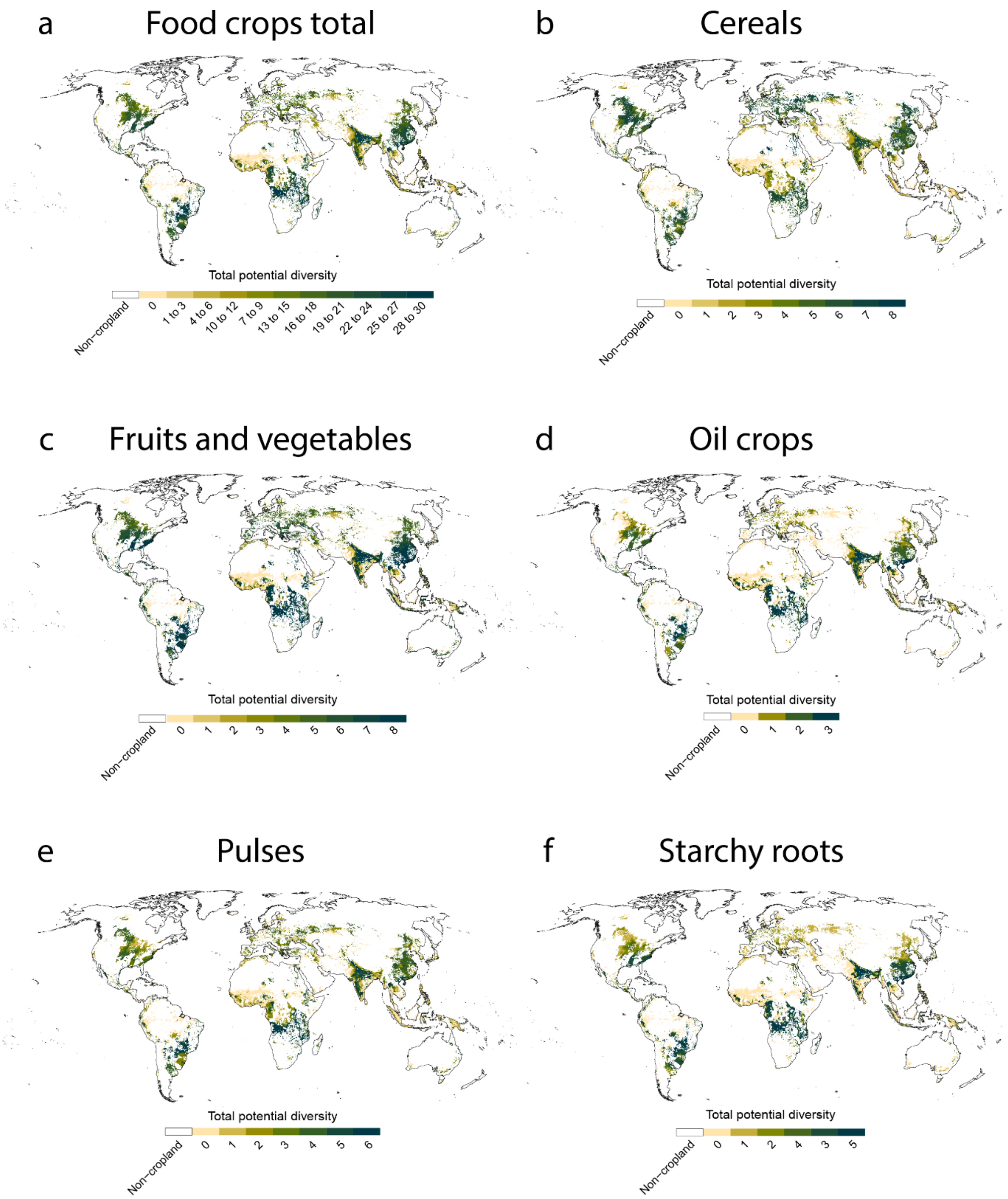

Supplementary Figure 3 Total potential food crop diversity under 3°C global warming. *Potential crop diversity was determined by projecting the extent of cropland within the crop-specific Safe Climatic Spaces (SCS) of the 30 analysed food crops on the current total cropland, regardless of the current cultivation area of each crop. Potential diversity is measured as the number of crops that could be cultivated in each location (grid cell) based on whether the crop-specific SCS covers the location under the warming level. In each panel, the category showing the highest value indicates the maximum potential diversity in that crop group. Panel a): food crops total; panels b) – e): crop groups (Supplementary Table 1). Coastline is from Natural Earth (naturalearthdata.com).*

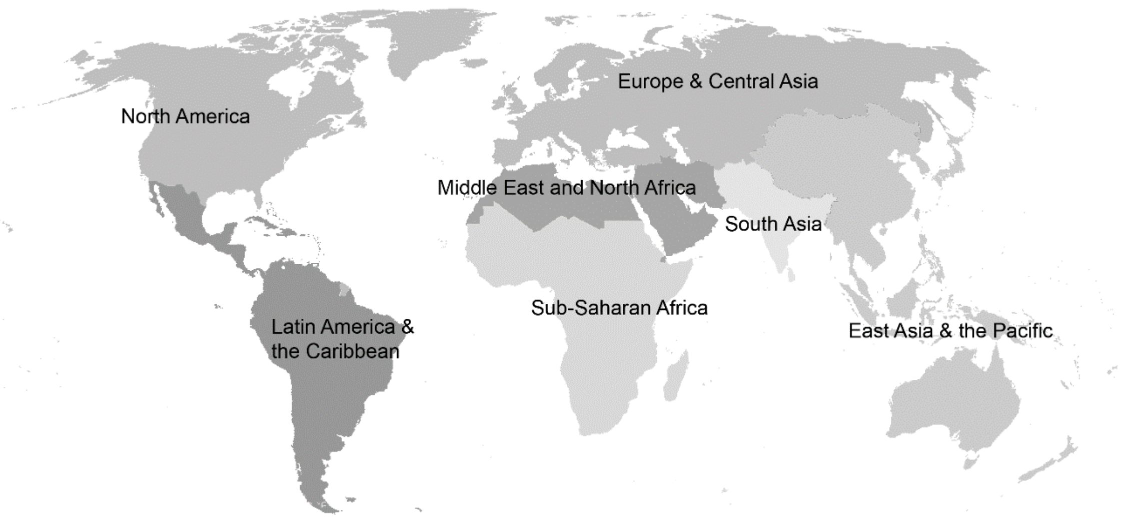

Supplementary Figure 4 World Bank regional division[2] used for regionally aggregating results. *Region boundaries are from Natural Earth (naturalearthdata.com).*

Supplementary Note 1

Uncertainty analysis with the SPAM 2005 and SPAM 2010 crop production datasets

Since the Safe Climatic Space (SCS) concept[3] defines the climatic niche for food crop production based on the baseline climatic extent of the current croplands, the spatial extent of the crop production data used might have a considerable impact on which climate conditions are classified within the SCS. To evaluate the impact of the geographical extent of the crop production data, we performed some of the analyses using two previous versions of the SPAM 2020 food production dataset[4]: SPAM 2005[5] and SPAM 2010[6], which represent crop production in the years 2005 and 2010, respectively. The cropland areas in the SPAM 2005 and SPAM 2010 datasets are more widely spatially distributed than those in the SPAM 2020 dataset. On the other hand, the global physical cropland area is the largest in SPAM 2020, at 12.9 million km^2, while in SPAM 2005, the global physical cropland area is 11.3 million km^2, and in SPAM 2010, it is 11.9 million km^2.

The SPAM 2005 and the SPAM 2010 datasets include 27 separate food crop types, whereas the SPAM 2020 dataset includes 30 food crop types. The food crops not included separately in the two previous SPAM datasets are tomato, onion, and citrus fruit. In the two previous datasets, the production of tomato and onion is allocated under the aggregate crop type 'vegetables' ('other vegetables' in SPAM 2020), and the production of citrus fruit under the aggregate crop type 'tropical fruit'[1]. The uncertainty analyses were performed for the 27 crop types found in the SPAM 2005 and SPAM 2010 data, and therefore, in the uncertainty analysis, the production of tomato and onion in SPAM 2020 were aggregated under the crop type 'vegetables', and the production of citrus fruit under the crop type 'tropical fruit'.

Examining the impacts of global warming on current crop production, Supplementary Table 7 shows the share of current cropland area where at least 25% (in brackets, 50% and 75%) of current production shifts outside the crop-specific SCS under each global warming level, using the SPAM 2005 data. Supplementary Table 8 shows a similar analysis using SPAM 2010 data. These results are similar to the main results (Figure 1, Supplementary Table 2), generally with a difference of 1 – 5 percentage points. Larger differences, up to 10 percentage points, are found in the Middle East and North Africa.

Supplementary Table 7 Cumulative share of cropland area where current crop production would be pushed into considerable risk under global warming levels using SPAM 2005 crop production data. *Considerable risk is defined as least 25% [50%, 75%] of the current production of the 27 food crops in SPAM 2005[5] data shifting outside the crop-specific Safe Climatic Spaces (SCS). "Cropland within SCS under all warming levels" shows the share of cropland area where less than 25% [50%, 75%] of current production would be pushed outside the crop-specific SCS under all warming levels.*

	**Cumulative share of cropland area where at least 25% of production shifts outside the SCS under warming level (%)**				**Cropland within SCS under all warming levels (%)**
**Region**	**1.5°C**	**2°C**	**3°C**	**4°C**	
Global	12 [8, 5]	19 [13, 10]	37 [30, 25]	51 [44, 38]	49 [56, 62]
East Asia & Pacific	11 [7, 5]	19 [14, 11]	37 [31, 27]	49 [41, 37]	51 [59, 63]
Europe & Central Asia	4 [3, 2]	5 [4, 3]	8 [6, 5]	15 [10, 8]	85 [90, 92]
Latin America & Caribbean	11 [8, 5]	16 [12, 8]	31 [27, 20]	45 [40, 34]	55 [60, 66]
Middle East & North Africa	39 [30, 22]	50 [40, 29]	65 [58, 48]	75 [69, 62]	24 [31, 38]
North America	7 [3, 2]	9 [4, 2]	12 [6, 4]	17 [10, 7]	83 [90, 93]
South Asia	16 [9, 6]	27 [17, 12]	62 [43, 34]	88 [78, 66]	12 [22, 34]
Sub-Saharan Africa	19 [10, 6]	31 [21, 16]	61 [55, 50]	77 [72, 68]	23 [28, 32]

Supplementary Table 8 Cumulative share of cropland area where current crop production would be pushed into considerable risk under global warming levels using SPAM 2010 crop production data. *Considerable risk is defined as least 25% [50%, 75%] of the current production of the 27 food crops in SPAM 2010[6] data shifting outside the crop-specific Safe Climatic Spaces (SCS). "Cropland within SCS under all warming levels" shows the share of cropland area where less than 25% [50%, 75%] of current production would be pushed outside the crop-specific SCS under all warming levels.*

	**Cumulative share of cropland area where at least 25% of production shifts outside the SCS under warming level (%)**				**Cropland within SCS under all warming levels (%)**
**Region**	**1.5°C**	**2°C**	**3°C**	**4°C**	
Global	12 [8, 5]	20 [14, 10]	38 [31, 25]	52 [45, 40]	48 [55, 60]
East Asia & Pacific	11 [7, 5]	19 [14, 11]	37 [31, 28]	49 [42, 39]	51 [58, 61]
Europe & Central Asia	6 [3, 2]	7 [4, 3]	9 [6, 4]	17 [11, 9]	83 [89, 91]
Latin America & Caribbean	9 [7, 5]	14 [11, 8]	31 [26, 19]	46 [41, 35]	54 [59, 65]
Middle East & North Africa	36 [28, 21]	45 [36, 27]	62 [55, 44]	74 [67, 58]	26 [33, 42]
North America	7 [3, 2]	9 [4, 3]	12 [7, 4]	17 [11, 8]	83 [89, 92]
South Asia	17 [9, 5]	29 [18, 11]	65 [49, 35]	90 [83, 71]	10 [17, 29]
Sub-Saharan Africa	18 [10, 6]	32 [22, 16]	62 [55, 50]	76 [70, 67]	24 [30, 33]

With regard to changes in cropland area within the SCSs of individual crops under projected climate conditions, the results produced using the SPAM 2020 (Supplementary Table 9), SPAM 2005 (Supplementary Table 10), and SPAM 2010 datasets (Supplementary Table 11) are generally similar, with differences of approximately 2–3 percentage points in the results for individual crops. However, for a few crops (yams, chickpea, lentil, plantain), there are considerably larger differences in the results (up to 22 percentage points with SPAM 2005 for chickpea and up to 20 percentage points with SPAM 2010 for chickpea). The largest disagreements in the results are found under 3°C and 4°C warming. The SPAM 2005 and SPAM 2010 results show overall slightly smaller decreases in cropland area within the SCS than the SPAM 2020 results.

Finally, for global changes in the potential food crop diversity of all 27 crops, the results produced using SPAM 2005 (Supplementary Table 12) and SPAM 2010 (Supplementary Table 13) are very similar to the main results in Supplementary Table 4. To conclude, the results produced using the SPAM 2005 and SPAM 2010 data are mostly similar to the main results produced using the SPAM 2020 data and support the main conclusions drawn from the main results. The differences in the results produced using the two datasets were the smallest for the globally aggregated results, as well as for the aggregated results of all crops, and the largest for the results for individual crops. The larger differences in results for individual crops and regions could be explained by changes in the actual distribution of the cultivation areas of each crop since the crop production datasets represent different years. Moreover, these differences could be explained by differences in the spatial extent of the crop production datasets. Therefore, the robustness of the results, especially at the level of individual crops, is impacted by the selected crop production data. The differences between the three crop production datasets result from improvements in the SPAM data production process[1], and we believe that results produced using the most recent crop production dataset, SPAM 2020, provide an improved estimate of shifts in the climatic niches of crops compared to results produced using the SPAM 2005 and the SPAM 2010 datasets.

Supplementary Table 9 Percentage net change in cropland area within the crop-specific Safe Climatic Space (SCS) of 27 food crops in SPAM 2020 data using SPAM 2005 and SPAM 2010 crop types. *The percentage change was calculated as the change in area within the SCS at the warming level compared to the cropland area within the SCS under baseline climate. 'Med' indicates the median estimate from 8 General Circulation Models (GCM), and '25th' and '75th' indicate the 25th and 75th percentiles of the GCM estimates, respectively. Tomato and onion production in SPAM 2020 data were aggregated into the crop type 'vegetables', and citrus fruit into the crop type 'tropical fruit' to facilitate comparison with results produced using the SPAM 2005[5] and SPAM 2010[6] crop types.*

	**Global warming (°C), measure**			
**Crop**	**1.5, med [25th, 75th]**	**2, med [25th, 75th]**	**3, med [25th, 75th]**	**4, med [25th, 75th]**
Wheat	-9 [-11, -8]	-15 [-19, -14]	-28 [-31, -25]	-38 [-42, -35]
Rice	-3 [-3, -2]	-7 [-11, -6]	-22 [-28, -18]	-40 [-51, -33]
Maize	-1 [-3, -1]	-7 [-11, -4]	-23 [-27, -17]	-37 [-44, -33]
Barley	-8 [-9, -7]	-13 [-14, -11]	-22 [-23, -19]	-30 [-33, -27]
Pearl millet	2 [1, 4]	1 [0, 3]	-16 [-22, -10]	-39 [-51, -31]
Small millet	-1 [-1, -1]	-4 [-6, -3]	-14 [-17, -10]	-30 [-40, -24]
Sorghum	3 [3, 4]	2 [-1, 4]	-10 [-13, -4]	-23 [-32, -17]
Other cereals	-3 [-3, -2]	-7 [-9, -5]	-20 [-24, -14]	-34 [-42, -31]
Potato	-9 [-10, -7]	-14 [-18, -13]	-28 [-31, -24]	-40 [-45, -36]
Sweet potato	0 [-3, 1]	-6 [-12, -3]	-23 [-29, -18]	-39 [-43, -32]
Yams	-1 [-2, 0]	-8 [-15, -5]	-34 [-41, -28]	-60 [-67, -51]
Cassava	0 [-1, 1]	-6 [-10, -3]	-28 [-32, -22]	-44 [-50, -36]
Other roots	-4 [-6, -2]	-10 [-16, -7]	-28 [-33, -22]	-42 [-46, -35]
Bean	-1 [-1, 0]	-3 [-5, -2]	-16 [-21, -12]	-31 [-39, -25]
Chickpea	-2 [-3, -1]	-6 [-7, -3]	-18 [-21, -14]	-33 [-35, -27]
Cowpea	2 [2, 3]	-1 [-4, 3]	-22 [-30, -15]	-46 [-54, -36]
Pigeon pea	-3 [-4, -1]	-9 [-14, -6]	-31 [-36, -25]	-50 [-51, -43]
Lentil	-11 [-14, -10]	-18 [-22, -16]	-31 [-32, -28]	-39 [-43, -37]
Other pulses	-6 [-7, -5]	-12 [-15, -10]	-26 [-30, -22]	-38 [-44, -35]
Soybean	-8 [-10, -6]	-16 [-20, -14]	-35 [-41, -28]	-49 [-52, -46]
Groundnut	4 [3, 5]	1 [-1, 4]	-14 [-18, -8]	-31 [-38, -22]
Coconut	6 [5, 7]	0 [-4, 3]	-26 [-32, -18]	-50 [-59, -39]
Banana	5 [4, 5]	3 [1, 6]	-13 [-18, -6]	-29 [-37, -19]
Plantain	1 [0, 2]	-6 [-12, -3]	-31 [-35, -24]	-47 [-49, -38]
Tropical fruit	2 [2, 3]	0 [-2, 3]	-12 [-15, -7]	-25 [-31, -18]
Temperate fruit	-1 [-2, 0]	-5 [-8, -3]	-20 [-24, -15]	-34 [-41, -29]
Vegetables	-1 [-1, 0]	-5 [-8, -3]	-19 [-23, -15]	-33 [-40, -28]

Supplementary Table 10 Percentage net change in cropland area within the crop-specific Safe Climatic Space (SCS) of the 27 food crops in SPAM 2005 data under global warming levels. *The percentage change was calculated as the change in area within the SCS at the warming level compared to the cropland area within the SCS under baseline climate. 'Med' indicates the median estimate from 8 General Circulation Models (GCM), and '25th' and '75th' indicate the 25th and 75th percentiles of the GCM estimates, respectively. Analogous to Supplementary Table 3 and Supplementary Table 9 but produced using SPAM 2005[5] crop production data.*

	**Global warming (°C), measure**			
**Crop**	**1.5, med [25th, 75th]**	**2, med [25th, 75th]**	**3, med [25th, 75th]**	**4, med [25th, 75th]**
Wheat	-8 [-9, -6]	-14 [-17, -13]	-26 [-28, -23]	-35 [-40, -33]
Rice	-3 [-4, -2]	-6 [-10, -5]	-18 [-24, -15]	-34 [-45, -29]
Maize	-1 [-2, 1]	-5 [-8, -2]	-19 [-23, -14]	-33 [-40, -30]
Barley	-8 [-9, -7]	-13 [-15, -13]	-26 [-28, -22]	-34 [-39, -31]
Pearl millet	2 [1, 3]	0 [-2, 3]	-14 [-19, -9]	-35 [-43, -27]
Small millet	-2 [-2, -1]	-4 [-6, -3]	-21 [-24, -14]	-36 [-47, -30]
Sorghum	4 [4, 5]	4 [1, 5]	-6 [-9, -1]	-18 [-24, -12]
Other cereals	-5 [-5, -4]	-8 [-9, -7]	-18 [-22, -14]	-33 [-41, -30]
Potato	-7 [-9, -6]	-13 [-16, -12]	-25 [-27, -22]	-33 [-39, -30]
Sweet potato	3 [0, 4]	0 [-4, 1]	-14 [-18, -9]	-29 [-35, -22]
Yams	9 [7, 10]	4 [-3, 6]	-24 [-33, -17]	-54 [-57, -44]
Cassava	2 [1, 3]	-2 [-6, 1]	-22 [-27, -16]	-39 [-43, -29]
Other roots	0 [-1, 1]	-5 [-9, -3]	-19 [-25, -15]	-34 [-36, -28]
Bean	0 [0, 0]	-2 [-4, -1]	-14 [-18, -10]	-27 [-34, -22]
Chickpea	6 [6, 8]	6 [4, 9]	-4 [-5, 3]	-14 [-14, -5]
Cowpea	3 [3, 4]	1 [-2, 4]	-20 [-26, -13]	-39 [-43, -30]
Pigeon pea	0 [-1, 2]	-4 [-8, -2]	-22 [-27, -17]	-42 [-45, -35]
Lentil	-3 [-6, -2]	-7 [-12, -6]	-18 [-19, -14]	-25 [-29, -21]
Other pulses	-3 [-4, -2]	-7 [-9, -5]	-19 [-22, -15]	-30 [-37, -26]
Soybean	-7 [-9, -6]	-14 [-18, -12]	-31 [-36, -26]	-44 [-51, -41]
Groundnut	5 [4, 6]	3 [1, 6]	-10 [-12, -3]	-23 [-31, -15]
Coconut	10 [8, 11]	2 [-7, 4]	-28 [-38, -22]	-55 [-59, -45]
Banana	6 [5, 6]	4 [2, 6]	-8 [-13, -4]	-25 [-35, -15]
Plantain	4 [3, 4]	-1 [-5, 2]	-20 [-25, -14]	-37 [-42, -29]
Tropical fruit	1 [0, 2]	-2 [-5, 0]	-14 [-17, -10]	-25 [-31, -20]
Temperate fruit	1 [1, 2]	-2 [-5, -1]	-16 [-20, -12]	-30 [-37, -25]
Vegetables	0 [0, 0]	-3 [-6, -2]	-16 [-19, -12]	-28 [-36, -24]

Supplementary Table 11 Percentage net change in cropland area within the crop-specific Safe Climatic Space (SCS) of the 27 food crops in SPAM 2010 data under global warming levels. *The percentage change was calculated as the change in area within the SCS at the warming level compared to the cropland area within the SCS under baseline climate. 'Med' indicates the median estimate from 8 General Circulation Models (GCM), and '25th' and '75th' indicate the 25th and 75th percentiles of the GCM estimates, respectively. Analogous to Supplementary Table 3 and Supplementary Table 9 but produced using SPAM 2010[6] crop production data.*

	**Global warming (°C), measure**			
**Crop**	**1.5, med [25th, 75th]**	**2, med [25th, 75th]**	**3, med [25th, 75th]**	**4, med [25th, 75th]**
Wheat	-8 [-10, -6]	-14 [-17, -12]	-27 [-30, -23]	-37 [-42, -34]
Rice	-3 [-4, -3]	-6 [-10, -5]	-19 [-25, -16]	-36 [-47, -31]
Maize	-1 [-3, 0]	-6 [-9, -3]	-20 [-24, -15]	-34 [-42, -31]
Barley	-7 [-8, -5]	-12 [-14, -11]	-23 [-25, -20]	-31 [-36, -28]
Pearl millet	3 [2, 4]	2 [0, 4]	-14 [-19, -9]	-35 [-43, -28]
Small millet	-3 [-4, -3]	-5 [-7, -4]	-20 [-23, -14]	-34 [-45, -29]
Sorghum	7 [7, 8]	7 [5, 9]	-2 [-4, 5]	-13 [-19, -6]
Other cereals	-3 [-3, -2]	-7 [-8, -5]	-19 [-23, -14]	-32 [-39, -29]
Potato	-8 [-10, -6]	-14 [-17, -12]	-27 [-29, -24]	-36 [-41, -33]
Sweet potato	0 [-1, 1]	-4 [-8, -2]	-20 [-24, -15]	-35 [-44, -31]
Yams	10 [8, 10]	6 [-1, 7]	-22 [-29, -14]	-51 [-56, -39]
Cassava	1 [0, 2]	-3 [-8, 0]	-24 [-29, -17]	-40 [-43, -30]
Other roots	-1 [-2, 1]	-6 [-11, -3]	-20 [-27, -16]	-35 [-37, -29]
Bean	-2 [-2, -2]	-5 [-7, -4]	-19 [-23, -15]	-32 [-39, -27]
Chickpea	2 [1, 4]	0 [0, 4]	-7 [-8, 0]	-13 [-17, -9]
Cowpea	5 [4, 6]	2 [0, 5]	-17 [-24, -10]	-40 [-44, -30]
Pigeon pea	-3 [-3, 0]	-7 [-12, -5]	-28 [-34, -23]	-45 [-47, -39]
Lentil	-3 [-5, -2]	-7 [-12, -5]	-19 [-20, -14]	-27 [-31, -23]
Other pulses	-2 [-2, -2]	-5 [-6, -4]	-14 [-17, -11]	-26 [-34, -22]
Soybean	-6 [-7, -5]	-14 [-17, -11]	-30 [-37, -24]	-45 [-52, -42]
Groundnut	6 [5, 7]	5 [2, 7]	-8 [-12, -2]	-22 [-30, -14]
Coconut	6 [5, 7]	2 [-2, 5]	-22 [-28, -14]	-47 [-55, -35]
Banana	5 [5, 5]	2 [1, 5]	-13 [-17, -7]	-30 [-39, -20]
Plantain	2 [2, 3]	-2 [-4, 0]	-17 [-20, -11]	-34 [-38, -23]
Tropical fruit	2 [1, 3]	-1 [-3, 2]	-11 [-15, -7]	-23 [-28, -17]
Temperate fruit	-1 [-2, 0]	-4 [-8, -2]	-18 [-23, -15]	-33 [-39, -29]
Vegetables	-2 [-3, -1]	-6 [-9, -5]	-21 [-24, -16]	-33 [-40, -29]

Supplementary Table 12 Global share of cropland in categories of change in potential crop diversity under global warming using SPAM 2005 data. *The change in potential diversity of the 27 food crops in SPAM 2005[5] data was calculated by comparing the potential diversity at the warming level to baseline potential diversity in 1990–2020 climate. "Cropland with emerging climatic potential" indicates that the area hosts marginal crop production in the baseline climate but would shift into the Safe Climatic Space (SCS) of at least one crop under the warming level. "Marginal in baseline and outside the SCS under warming level" indicates that the area hosts marginal production in the baseline climate and is not within the SCS of any crop under the warming level.*

	**Share of global cropland in category under global warming levels (%)**			
**Potential diversity change compared to baseline (%)**	**1.5°C**	**2°C**	**3°C**	**4°C**
-100	1.0	3.4	14.2	26.6
-99.99 to -75	1.0	2.3	5.3	5.6
-75 to -50	2.3	3.8	3.9	4.8
-50 to -25	7.1	9.7	9.2	8.1
-25 to 0	27.2	27.5	21.0	14.0
no change	23.4	12.7	10.0	9.9
0 to +25	26.5	26.4	19.5	15.1
+25 to +50	7.6	9.0	9.3	8.3
+50 to +75	1.9	2.7	4.2	4.3
+75 to +100	1.1	1.6	2.4	2.4
Cropland with emerging climatic potential	0.3	0.3	0.4	0.5
Marginal in baseline and outside the SCS under warming level	0.6	0.5	0.4	0.4

Supplementary Table 13 Global share of cropland in categories of change in potential crop diversity under global warming using SPAM 2010 data. *The change in potential diversity of the 27 food crops in SPAM 2010[6] data was calculated by comparing the potential diversity at the warming level to baseline potential diversity in 1990–2020 climate. "Cropland with emerging climatic potential" indicates that the area hosts marginal crop production in the baseline climate but would shift into the Safe Climatic Space (SCS) of at least one crop under the warming level. "Marginal in baseline and outside the SCS under warming level" indicates that the area hosts marginal production in the baseline climate and is not within the SCS of any crop under the warming level.*

	**Share of global cropland in category under global warming levels (%)**			
**Potential diversity change compared to baseline (%)**	**1.5°C**	**2°C**	**3°C**	**4°C**
-100	1.0	3.2	13.1	26.2
-99.99 to -75	1.0	2.8	6.8	7.4
-75 to -50	2.7	4.3	5.2	5.7
-50 to -25	8.3	10.5	9.8	7.3
-25 to 0	26.7	27.0	18.6	12.6
no change	23.4	12.6	9.9	9.3
0 to +25	25.7	25.6	19.9	15.3
+25 to +50	7.5	9.3	10.3	8.8
+50 to +75	1.9	2.3	3.6	4.4
+75 to +100	1.1	1.5	2.0	2.2
Cropland with emerging climatic potential	0.2	0.3	0.4	0.4
Marginal in baseline and outside the SCS under warming level	0.5	0.5	0.4	0.4

Uncertainty analysis with Safe Climatic Space defined based on crop calendar

The Safe Climatic Space (SCS) concept[3] defines the climatic niche of food crops based on average annual climate parameter values, utilizing the Holdridge Life Zones framework[7]. Therefore, it has a limited ability to represent the seasonal variability of climate conditions. However, the seasonal variability in climate strongly influences cropping patterns, for example, in regions with monsoon rainfall and multiple cropping seasons. For instance, in Latin America, soybean is cultivated during the rainy winter, and maize might be planted early in the spring when the drier season begins[8]. Therefore, the climatic niches of these crops in Latin America are not similar in reality, but the distinction cannot be represented using the original annual SCS approach. To examine the impact of the seasonality of climate conditions on the results of this study, we performed additional analyses in which crop-specific SCSs were defined based on a local (grid cell level) crop calendar. The additional analyses were limited to two crops: maize and soybean. The seasonal analysis assumes that crop calendars are fixed under all warming levels and therefore does not consider the effects of locally shifting crop calendars as a climate change adaptation method.

We performed the analyses considering the crop calendars of maize and soybean using the GGCMI Phase 3 crop calendar dataset[9]. These two crops were selected because, for them, the spatial coverage of the crop calendar data was the largest out of the crops that match the crops in the SPAM 2020[4] crop production data. Using both irrigated and rainfed crop calendars[9], we created monthly cropping season filter rasters for maize and soybean. Then, at the grid cell level, we selected only the months of temperature and precipitation data[10] that fell within the cropping season of each crop for further calculation. Next, we applied the same crop-specific SCS calculation steps as described in the Methods section and performed analyses of the changes in cropland area within the SCS for the current production areas of these crops as well as for the total cropland area of all crops. We compared the results from these analyses to the corresponding results using the original annual SCS method. The comparison was performed for the overlapping parts of the total cropland area, maize production area, and soybean production area between the seasonal and the annual SCS methods.

At the global scale, seasonal and annual SCS approaches produced similar results for the share of current crop production that would shift outside the SCS (Supplementary Table 14, Supplementary Table 15). For maize, at the regional scale, the results obtained using the two approaches are similar under 1.5°C to 2°C global warming, but larger (> 10 percentage points) differences start to emerge under 3°C warming (Supplementary Table 15). For soybean, 10 percentage points or larger differences in results are seen already under 1.5°C warming in East Asia and the Pacific and in Sub-Saharan Africa, but in other regions, larger differences are observed only under 3°C and higher warming (Supplementary Table 14). For both crops, the annual approach generally shows larger adverse impacts on current crop production in East Asia and the Pacific, the Middle East and North Africa, South Asia, and Sub-Saharan Africa, i.e., regions with multiple cropping[8] and monsoon rainfall. On the other hand, the seasonal approach generally shows larger adverse impacts on Europe and Central Asia and North America.

When shifts in the cropland within the SCS are projected on the total cropland of all crops, globally, the seasonal and annual SCS approaches show very similar results (Supplementary Table 16). Under 3°C warming, areas where the two approaches disagree are distributed across world regions (Supplementary Figure 5). However, the seasonal approach estimates more area within the SCS in the equatorial region. For both soybean and maize, these areas

within the seasonal SCS but outside the annual SCS partially overlap current multiple cropping areas[8] and monsoon regions. On the other hand, the annual approach estimates more cropland within the SCS in Europe and Central Asia, Southern America, and Central and Southern Africa. The areas where the two approaches agree and disagree are distributed similarly for soybean and maize.

Overall, the annual and seasonal SCS approaches produce similar results at the regional scale under 1.5°C to 2°C global warming and at the global scale under all warming levels. The largest uncertainties are observed in multiple cropping and monsoon rainfall regions. There are at least two possible explanations for the increasing difference in the results between the two approaches under 3°C and higher warming. First, under higher warming levels, the overall greater change in climate conditions might contribute to the larger difference between the annual and seasonal SCSs. Second, since the seasonal analysis applied a temporally fixed crop calendar, climate conditions that would fit the SCS of a crop might temporally shift outside the local growing season (defined at the grid cell level) in the seasonal analysis. Therefore, the seasonal approach might classify some locations outside the SCS of a crop even though suitable climate conditions would exist at a different time of the year.

Supplementary Table 14 Median share of the current soybean production outside the crop-specific Safe Climatic Space (SCS) under global warming, using the original, annual SCS method and the seasonal SCS method based on crop calendar. *Share of production was calculated from production data in metric tons. 'Ann' columns show results using the annual method and 'Seas' columns results using the seasonal method. 'Tot major prod' is the major production (contributing to the highest 95% of the global total in metric tons) in the region. These values differ between the SCS methods because grid cells contributing to the major production areas are found by assigning all production areas to climatic bins using precipitation and PET data and then choosing the climatic bins that include the highest ≤ 95% of production[3]. NA indicates that there is currently no major production in the region, and therefore, the effects on current production cannot be calculated.*

Share of current production outside SCS (%) under warming level										
	1.5°C		2°C		3°C		4°C		Tot major prod (1000 mtons)	
**Region**	**Ann**	**Seas**	**Ann**	**Seas**	**Ann**	**Seas**	**Ann**	**Seas**	**Ann**	**Seas**
Global	10	9	14	14	28	27	36	34	323047	322590
East Asia & Pacific	12	2	19	4	33	8	38	8	15939	16894
Europe & Central Asia	38	34	39	38	32	59	33	84	2590	2013
Latin America & Caribbean	5	7	10	11	29	21	44	27	180874	179502
Middle East & North Africa	25	NA	19	NA	14	NA	35	NA	3	0
North America	16	13	20	22	22	43	19	56	112000	113498
South Asia	4	5	15	9	60	6	98	4	9780	9317
Sub-Saharan Africa	31	8	44	17	42	21	44	21	1862	1366

Supplementary Table 15 Median share of the current maize production outside the crop-specific Safe Climatic Space (SCS) under global warming, using the original, annual SCS method and the seasonal SCS method based on crop calendar. *Share of production was calculated from production data in metric tons. 'Ann' columns show results using the annual method and 'Seas' columns results using the seasonal method. 'Tot major prod' is the major production (contributing to the highest 95% of the global total in metric tons) in the region. These values differ between the SCS methods because grid cells contributing to the major production areas are found by assigning all production areas to climatic bins using precipitation and PET data and then choosing the climatic bins that include the highest ≤ 95% of production[3]. NA indicates that there is currently no major production in the region, and therefore, the effects on current production cannot be calculated.*

**Share of current production outside SCS (%) under warming level**										
	**1.5°C**		**2°C**		**3°C**		**4°C**		**Tot major prod (1000 mtons)**	
**Region**	**Ann**	**Seas**	**Ann**	**Seas**	**Ann**	**Seas**	**Ann**	**Seas**	**Ann**	**Seas**
Global	4	4	6	6	12	12	21	21	1108713	1103836
East Asia & Pacific	6	3	8	4	14	6	19	7	287784	291954
Europe & Central Asia	2	7	3	10	9	27	21	51	136184	132361
Latin America & Caribbean	3	4	6	7	14	11	31	14	192941	185057
Middle East & North Africa	6	1	5	1	5	1	5	2	5658	9011
North America	2	1	2	3	3	9	6	23	368823	373579
South Asia	11	9	19	19	39	27	79	36	39348	39239
Sub-Saharan Africa	12	11	24	15	34	24	41	36	77976	72636

Supplementary Table 16 Median net change in the global total cropland area within the crop-specific Safe Climatic Spaces (SCS) of soybean and maize under global warming. *The 'Ann' columns show the net change using the original annual SCS method, and the 'Seas' columns show the net change using the seasonal SCS method based on the crop calendar. Net change was calculated as the percentage change in the total cropland area within the SCS (regardless of where the crop is currently grown) from baseline climate conditions to a warming level. The total cropland area refers to the current total cropland of the 46 crop types in the SPAM 2020 data[4].*

**Share of current total cropland outside SCS (%) under warming level**								
	**1.5°C**		**2°C**		**3°C**		**4°C**	
**Crop**	**Ann**	**Seas**	**Ann**	**Seas**	**Ann**	**Seas**	**Ann**	**Seas**
Soybean	10	9	14	14	28	27	36	34
Maize	4	4	6	6	12	12	21	22

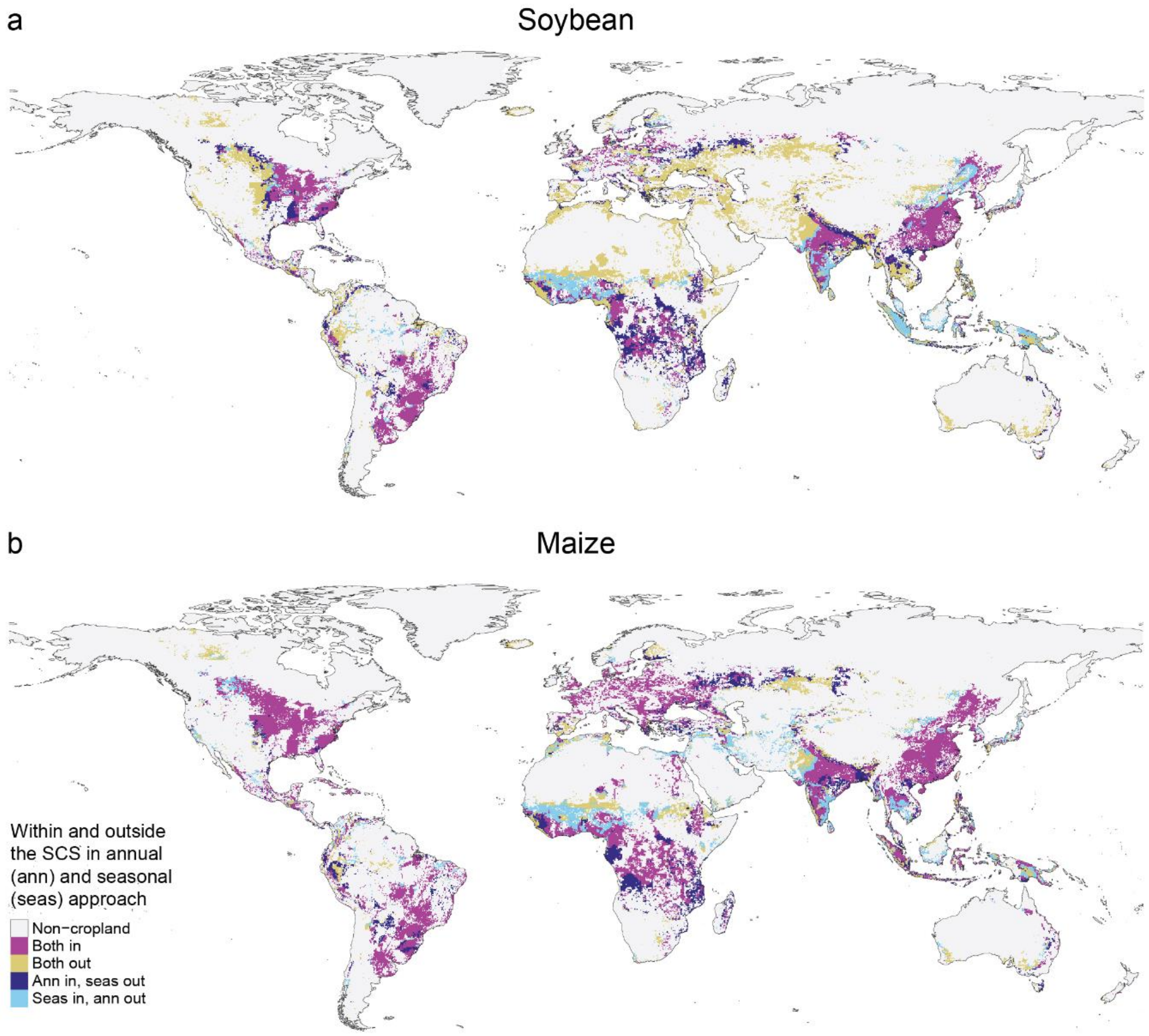

Supplementary Figure 5 Current cropland within and outside the Safe Climatic Space (SCS) under 3°C global warming using the original, annual SCS approach and the seasonal SCS approach. *The annual SCS approach uses climate parameter data from the whole year whereas the seasonal approach considers the crop calendar and only uses climate parameter data within the cropping season. Panels: a) soybean, b) maize. Coastline is from Natural Earth (naturalearthdata.com).*

Supplementary Note 2

Changes in potential crop diversity within elevation and latitude zones

We measured changes in potential crop diversity for food crops in total within elevation and latitude zones using the same calculation steps as for summarization within geographical regions (Methods). The elevation zones were created using the HydroSHEDS v1[11] Void-filled Digital Elevation Model (DEM) with a 30 arc-second resolution. The DEM was upscaled to 5 arc-minute resolution using the *bilinear* method of the *resample* function of the *terra* R package[12] and then classified into four elevation regions: less than 800 m (86.1% of current cropland), 800 m to 1500 m (9.4%), 1500 m to 2500 m (3.9%), and more than 2500 m (0.6%). The latitude regions were delineated by creating a 5-arc minute resolution raster with latitude as the cell value and classifying the raster into three zones: 30° to 90° N (mid-to-high latitudes in the Northern Hemisphere, 40.6% of current cropland), 40° N to 40° S (equatorial region, 55.5%), and 30° to 90° S (mid-to-high latitudes in the Southern Hemisphere, 3.9%).

Both elevation and latitude zone rasters were polygonised to allow calculating zonal statistics of the change in potential diversity.

Within the elevation zones, the zone with less than 800 m elevation would experience the largest and most severe decrease in potential food crop diversity, and the zone with elevation of more than 2500 m the smallest and the mildest decrease (Supplementary Figure 6). The pattern of change in the lowest elevation zone closely aligns with the global change shown in Figure 5. In contrast, the highest elevation zone exhibited changes similar to those found in North America (Figure 5), showing an increase in potential diversity across most of the current cropland area under all warming levels. The highest elevation zone experiences the largest increases in cropland area with emerging climatic potential (4% to 10% under 1.5°C to 4°C global warming, respectively). It is worth noting that this zone also encompasses a larger share of the baseline marginal cropland land area than other elevation zones and covers only 0.6% of the current total cropland area.

Within latitude zones, the equatorial region (30° N to 30° S) faces more extensive and severe loss of potential diversity than the mid-to-high latitude zones (30° to 90°) in the Northern and Southern Hemispheres (Supplementary Figure 6). In the equatorial region, all potential diversity (-100%) would be lost on more than 25% of the cropland area if global warming exceeded 2°C. On the other hand, in the mid-to-high latitudes in the Northern Hemisphere, there is stronger increase in potential crop diversity than in the other two latitude zones. There, the potential crop diversity would increase by more than 50% compared to the baseline potential diversity on 7% to 13% of the cropland area under 1.5°C to 4°C global warming, respectively. In the other two latitude zones, a greater than 50% increase in potential diversity would be found only on 1–2% of the current cropland area. In the mid-to-high latitudes in the Southern Hemisphere, the changes in potential diversity are milder than those in the other latitude zones: on 90% to 66% of the cropland area (under 1.5°C to 4°C global warming, respectively), the potential crop diversity would increase or decrease less than 25% of the cropland area under all warming levels.

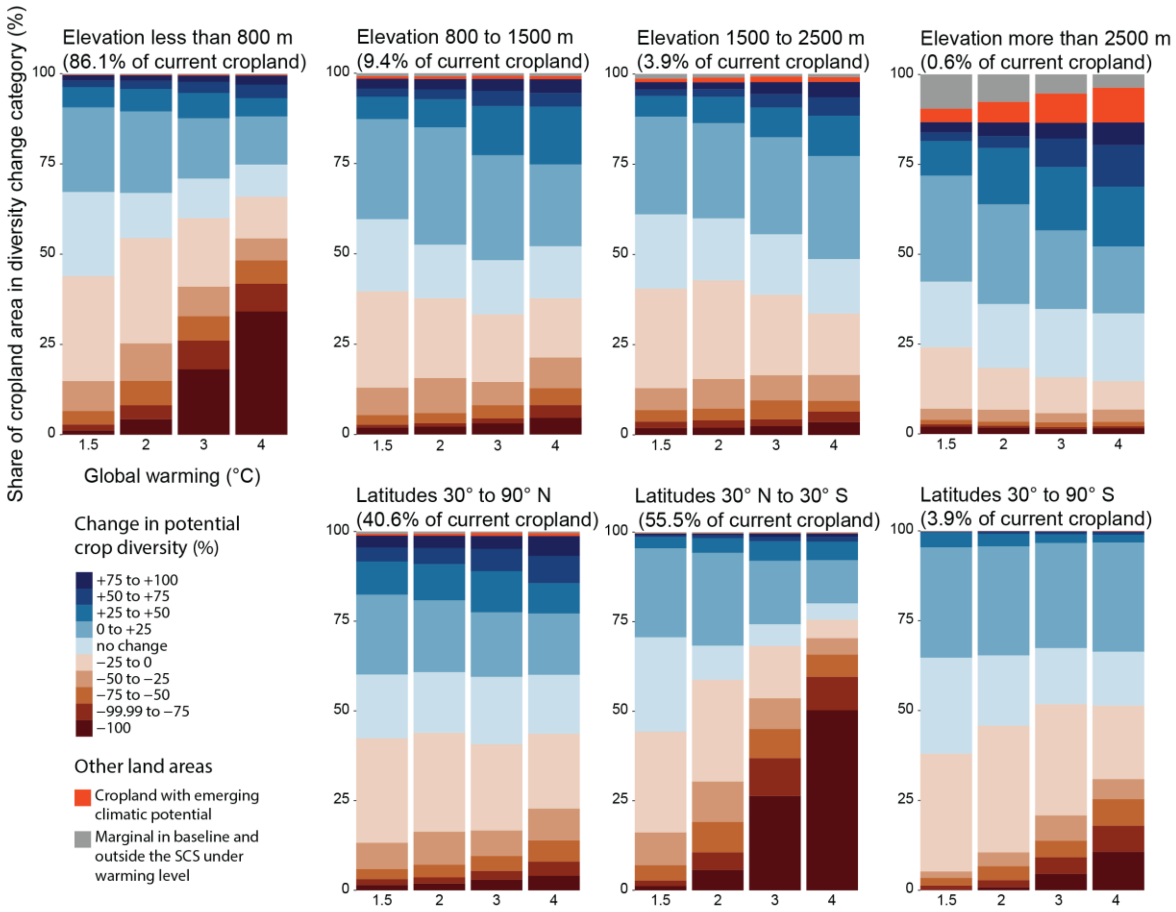

Supplementary Figure 6 Changes in the potential food crop diversity in elevation and latitude zones. *Stacked bars show the share of current cropland area in categories of potential diversity change under four global warming levels from 1.5°C to 4°C. The change in potential diversity is measured as the percentage change in the number of crops that could be cultivated at each location given the temperature and moisture conditions from the baseline climate conditions to the global warming level. The number of crops that could be cultivated in a location is determined by the geographical extent of the crop-specific Safe Climatic Spaces (SCS), regardless of the current cultivation area of the individual crops. For an individual crop, a location is defined within the SCS if at least half of the eight General Circulation Models indicate this. "Marginal in baseline and outside the SCS under warming level" indicates that the location currently hosts marginal crop production and does not shift into the SCS of any crop under the warming levels. "Cropland with emerging climatic potential" indicates that the location currently hosts marginal crop production but shifts into the SCS of at least one crop under a warming level.*

Supplementary references

1. Yu, Q. *et al.* A cultivated planet in 2010 – Part 2: The global gridded agricultural-production maps. *Earth System Science Data* **12**, 3545–3572 (2020).
2. The World Bank Group. World Bank Country and Lending Groups – World Bank Data Help Desk. https://datahelpdesk.worldbank.org/knowledgebase/articles/906519-world-bank-country-and-lending-groups.

3. Kummu, M., Heino, M., Taka, M., Varis, O. & Viviroli, D. Climate change risks pushing one-third of global food production outside the safe climatic space. *One Earth* **4**, 720–729 (2021).
4. IFPRI. Global Spatially-Disaggregated Crop Production Statistics Data for 2020 Version 1.0. Harvard Dataverse https://doi.org/10.7910/DVN/SWPENT (2024).
5. IFPRI & IIASA. Global Spatially-Disaggregated Crop Production Statistics Data for 2005 Version 3.2. Harvard Dataverse https://doi.org/10.7910/DVN/DHXBJX (2016).
6. IFPRI. Global Spatially-Disaggregated Crop Production Statistics Data for 2010 Version 2.0. Harvard Dataverse https://doi.org/10.7910/DVN/PRFF8V (2019).
7. Holdridge, L. R. Determination of World Plant Formations from Simple Climatic Data. *Science* **105**, 367–368 (1947).
8. Waha, K. *et al.* Multiple cropping systems of the world and the potential for increasing cropping intensity. *Global Environmental Change* **64**, 102131 (2020).
9. Jägermeyr, J., Müller, C., Minoli, S., Ray, D. & Siebert, S. GGCMI Phase 3 crop calendar. Zenodo https://doi.org/10.5281/zenodo.5062513 (2021).
10. Fick, S. E. & Hijmans, R. J. WorldClim 2: new 1-km spatial resolution climate surfaces for global land areas. *International Journal of Climatology* **37**, 4302–4315 (2017).
11. Lehner, B., Verdin, K. & Jarvis, A. New Global Hydrography Derived From Spaceborne Elevation Data. *Eos, Transactions American Geophysical Union* **89**, 93–94 (2008).
12. Hijmans, R. J. R package terra: Spatial Data Analysis. (2023).
