# Supplementary material for: Climate change threatens crop diversity at low latitudes
Source: Nat Food. 2025 Mar 4;6(4):331–42. doi: 10.1038/s43016-025-01135-w (PMC12018264; doi:10.1038/s43016-025-01135-w)
Supplement: Supplementary file 2 — Reporting Summary [file 43016_2025_1135_MOESM2_ESM.pdf]

Corresponding author(s): Sara Heikonen, Matti Kummu

Last updated by author(s): Feb 4, 2025

Reporting Summary

Nature Portfolio wishes to improve the reproducibility of the work that we publish. This form provides structure for consistency and transparency in reporting. For further information on Nature Portfolio policies, see our Editorial Policies and the Editorial Policy Checklist.

Statistics

For all statistical analyses, confirm that the following items are present in the figure legend, table legend, main text, or Methods section.

n/a	Confirmed	
☐	☒	The exact sample size (*n*) for each experimental group/condition, given as a discrete number and unit of measurement
☒	☐	A statement on whether measurements were taken from distinct samples or whether the same sample was measured repeatedly
☒	☐	The statistical test(s) used AND whether they are one- or two-sided<br>*Only common tests should be described solely by name; describe more complex techniques in the Methods section.*
☒	☐	A description of all covariates tested
☒	☐	A description of any assumptions or corrections, such as tests of normality and adjustment for multiple comparisons
☐	☒	A full description of the statistical parameters including central tendency (e.g. means) or other basic estimates (e.g. regression coefficient) AND variation (e.g. standard deviation) or associated estimates of uncertainty (e.g. confidence intervals)
☒	☐	For null hypothesis testing, the test statistic (e.g. *F*, *t*, *r*) with confidence intervals, effect sizes, degrees of freedom and *P* value noted<br>*Give P values as exact values whenever suitable.*
☒	☐	For Bayesian analysis, information on the choice of priors and Markov chain Monte Carlo settings
☒	☐	For hierarchical and complex designs, identification of the appropriate level for tests and full reporting of outcomes
☒	☐	Estimates of effect sizes (e.g. Cohen's *d*, Pearson's *r*), indicating how they were calculated

Our web collection on statistics for biologists contains articles on many of the points above.

Software and code

Policy information about availability of computer code

Data collection	No software was used.
Data analysis	Custom code was used to download climate parameter data and to perform the analyses in this study. The code includes scripts in Matlab (version 9.14.0.2239454 [R2023a]) and R (version 4.4.1) programming languages and the code is available in Zenodo: https://doi.org/10.5281/zenodo.14804349.

For manuscripts utilizing custom algorithms or software that are central to the research but not yet described in published literature, software must be made available to editors and reviewers. We strongly encourage code deposition in a community repository (e.g. GitHub). See the Nature Portfolio guidelines for submitting code & software for further information.

Data

Policy information about availability of data

All manuscripts must include a data availability statement. This statement should provide the following information, where applicable:

- Accession codes, unique identifiers, or web links for publicly available datasets
- A description of any restrictions on data availability
- For clinical datasets or third party data, please ensure that the statement adheres to our policy

All analyses in this study were performed using openly available, external datasets, described in the manuscript in Methods, Supplementary note 1 and Supplementary note 2. The data from this study will be made openly available upon publication. The external datasets are listed in following:

- Temperature and precipitation for the following historical periods: 1970–2000 (30-year averages of monthly tmax, tmin, tmean, and prec) and 1990–2020 (monthly tmax, tmin, and prec), and for the following future periods, Shared Socioeconomic Pathways (SSP), and General Circulation Models (GCM): SSP1-2.6 periods 2021–2040 and 2041–2060, SSP2-4.5 periods 2041–2060 and 2061–2080, SSP3-7.0 periods 2061–2080 and 2081–2100, and SSP5-8.5 periods 2061–2080 and 2081–2100 (20-year averages of monthly tmax, tmin, and prec), all for the GCMs BCC-CSM2-MR, CNRM-CM6-1, CNRM-ESM2-1, canesm5, IPSL-CM6A-LR, MIROC-ES2L, MIROC6, and MRI-ESM2-0: WorldClim2, https://www.worldclim.org/data/index.html
- Crop specific production data (metric tons) and physical cropland area (hectares): SPAM 2020 v1.0 Global data, https://dataverse.harvard.edu/dataset.xhtml?persistentId=doi:10.7910/DVN/SWPENT#, SPAM 2010 v2.0 Global Data, https://dataverse.harvard.edu/dataset.xhtml?persistentId=doi:10.7910/DVN/PRFF8V#, and SPAM 2005 v3.2 Global Data, https://dataverse.harvard.edu/dataset.xhtml?persistentId=doi:10.7910/DVN/DHXBJX#
- Crop calendar (sowing date and harvest date) for maize and soybean, rainfed and irrigated: GGCMI Phase 3 crop calendar, https://doi.org/10.5281/zenodo.5062513
- Digital Elevation Model: HydroSHEDS v1 Void filled Digital elevation Model, https://data.hydrosheds.org/file/hydrosheds-v1-dem/hyd_glo_dem_30s.zip
The data produced in this study is openly available in Zenodo: https://doi.org/10.5281/zenodo.14801623. This data includes source data tables and images for all Figures, Extended Data Figures and Supplementary Figures as well as raster files of other outputs generated in this study.

Human research participants

Policy information about studies involving human research participants and Sex and Gender in Research.

Reporting on sex and gender	Human participants were not involved in this study.
Population characteristics	Human participants were not involved in this study.
Recruitment	Human participants were not involved in this study.
Ethics oversight	Human participants were not involved in this study.

Note that full information on the approval of the study protocol must also be provided in the manuscript.

Field-specific reporting

Please select the one below that is the best fit for your research. If you are not sure, read the appropriate sections before making your selection.

☐ Life sciences ☐ Behavioural & social sciences ☒ Ecological, evolutionary & environmental sciences

For a reference copy of the document with all sections, see nature.com/documents/nr-reporting-summary-flat.pdf

Ecological, evolutionary & environmental sciences study design

All studies must disclose on these points even when the disclosure is negative.

Study description	In this study, we assess the future climatic suitability of current croplands for 30 major food crops as well as quantify changes in potential food crop diversity across global croplands at 1.5°C to 4°C global warming. We utilized global gridded data on temperature and precipitation, crop production and physical cropland area, as well as crop calendars. The analyses is based on defining the climatic niches of individual food crops and projecting geographic shifts in the niches in future climate conditions.
Research sample	The sample of studied crops includes the 30 food crops found in SPAM 2020 data (27 food crops in Supplementary analysis with SPAM 2010 and SPAM 2005 data). See Data section for download links. This data was selected to maximize the number of crops to study, because these datasets were to our knowledge the most recent, spatially extensive, covered the largest number of food crops, and had the highest spatial resolution available at the time of selecting crop production data to use. The eight General Circulation Models where the climate parameter data in this study comes from were selected based on their use in Kummu et al. (2021), because we adapt method and code for defining climatic suitability for individual crops from Kummu et al. (2021). Crop calendar data was used for a sensitivity analysis only for maize and soybean because of the limited spatial coverage of crop calendar data for other studied crops.
Sampling strategy	The sample size for crop production data was limited by the availability of external datasets. The sample of General Circulation Models was chosen to keep consistency with Kummu et al. (2021) whose method we applied for defining suitable climate conditions for food crops. The sample size of sensitivity analysis was limited to maize and soybean due to limited availability of crop calendar data with large spatial coverage for other studied crops.
Data collection	The analyses in this study were performed using existing, openly available datasets, hence no data was collected for the study.
Timing and spatial scale	The external datasets were downloaded during 2021-2024. All data were of global extent. The crop production and physical cropland area data sets represent years 2020 (SPAM 2020), 2010 (SPAM 2010), and 2005 (SPAM 2005). The historical climate parameter data represent years 1970-2000 and 1990-2020, and the future climate data represent the following years and Shared Socioeconomic Pathways (SSP): SSP1-2.6 periods 2021–2040 and 2041–2060, SSP2-4.5 periods 2041–2060 and 2061–2080, SSP3-7.0 periods 2061–2080 and 2081–2100, and SSP5-8.5 periods 2061–2080 and 2081–2100. Crop calendar data was published in 2021 and the digital elevation model data in 2008.

Data exclusions	No data were excluded from the analysis.
Reproducibility	The reproducibility of the results has been ensured by making the full analysis code openly available, and by only using openly available data as input in the analysis. The input data are described above in the Data section as well as in the Methods and Data availability sections in the manuscript. In addition, the data generated in this study is openly available in Zenodo: xxx.
Randomization	Randomization was not relevant in this study because the methods do not include modeling or measuring relationships between variables.
Blinding	Blinding was not relevant because the study uses openly available, external datasets.

Did the study involve field work? ☐ Yes ☒ No

Reporting for specific materials, systems and methods

We require information from authors about some types of materials, experimental systems and methods used in many studies. Here, indicate whether each material, system or method listed is relevant to your study. If you are not sure if a list item applies to your research, read the appropriate section before selecting a response.

Materials & experimental systems

n/a	Involved in the study	
☒	☐	Antibodies
☒	☐	Eukaryotic cell lines
☒	☐	Palaeontology and archaeology
☒	☐	Animals and other organisms
☒	☐	Clinical data
☒	☐	Dual use research of concern

Methods

n/a	Involved in the study	
☒	☐	ChIP-seq
☒	☐	Flow cytometry
☒	☐	MRI-based neuroimaging
